# CEBPB-high dormant tumor cells drive immune evasion via S100A8 orchestrated tumor-associated macrophages reprogramming

**DOI:** 10.7150/thno.124789

**Published:** 2026-02-26

**Authors:** Jin Bai, Huilan Su, Shunxi Wang, Yang Dong, Yong Gao

**Affiliations:** 1 Department of Oncology, Shanghai East Hospital, Tongji University School of Medicine, 150 Jimo Road, Pudong District, Shanghai, 200120, China.; 2 Cancer Center of Daping Hospital, Army Medical University, Chongqing, 400037, China.; 3 Research Center for Translational Medicine, Cancer Stem Cell Institute, Shanghai East Hospital, Tongji University School of Medicine, 150 Jimo Road, Pudong District, Shanghai, 200120, China.; 4 Institute of Pathology and Southwest Cancer Center, Southwest Hospital, Third Military Medical University (Army Medical University), and The Key Laboratory of Tumor Immunopathology, The Ministry of Education of China, Chongqing, 400038, China; 5 Chongqing Institute of Advanced Pathology, Jinfeng Laboratory, Chongqing, 400039, China.

**Keywords:** Dormant tumor cells, macrophages, immune checkpoint blockade, CEBPB, S100A8

## Abstract

**Rationale:**

Triple negative breast cancer (TNBC) poorly responds to immune checkpoint blockade (ICB) therapy. Dormant tumor cells are recognized as immunotherapy-resistant reservoirs that may lead to tumor relapse, although the underlying mechanisms remain to be fully elucidated.

**Methods:**

Public scRNA-seq data was employed to identify dormant tumor cells in TNBC patients receiving ICB therapy. A TetOn-H2BeGFP system was used to label and track dormant tumor cells both *in vivo* and *in vitro*. CCK-8, colony formation, and PI staining assay were performed to identify CEBPB as a key factor in tumor dormancy maintenance. The role of CEBPB in immune evasion was evaluated by macrophage and CD8^+^ T cell proportions in tumors, via flow cytometry, immunohistochemistry, and multiplex immunofluorescence assays. RNA-seq and ChIP-seq were further employed to identify downstream targets of CEBPB, and ChIP-qPCR, qPCR, and Western blot were used to further validate these results.

**Results:**

We demonstrated that dormant tumor cells were resistant to ICB and resided within an immunosuppressive niche, characterized by increased M2 macrophages and reduced CD8^+^ T cell infiltration. CEBPB was identified as highly expressed in dormant tumor cells, where it maintained tumor dormancy by transcriptionally activating cell cycle negative regulators, particularly CCNG2. Notably, high CEBPB expression orchestrated a tumor-supportive microenvironment with macrophage recruitment, M2 macrophage polarization, and T cell suppression. Mechanistically, S100A8 was recognized as a key transcriptional target of CEBPB to promote M2 macrophage polarization. Targeting either CEBPB or S100A8 could overcome ICB resistance and remodel the tumor microenvironment.

**Conclusions:**

Our study demonstrate a mechanistic link between tumor dormancy and immune evasion, highlighting the CEBPB-S100A8 axis as a promising therapeutic target to potentiate ICB efficacy in TNBC.

## Introduction

Triple negative breast cancer (TNBC) is the most aggressive subtype of breast cancers and is associated with the worst prognosis 1. Given the absence of appropriate drug targets such as HER2, ER or PR, chemotherapy remains the only systemic treatment option for TNBC patients, limiting their therapeutic choices 2. Recently, immune checkpoint blockade (ICB) therapy, a highly promising drug, has been approved for TNBC treatment. However, only a few patients benefit from ICB in either early-stage or advanced TNBC 3,4. This highlights the critical need to elucidate mechanisms underlying immunotherapy resistance in order to improve treatment efficacy.

Dormant tumor cells constitute a crucial yet heterogeneous part of tumor cells, which are capable of evading immune surveillance 5,6. These tumor cells can reside an immune-privileged niche to ensure survival or maintain dormancy via limiting T-cell infiltration and increasing the presence of tumor-protective fibroblasts, tumor-associated macrophages (TAMs), or antigen-presenting-deficient dendritic cells 7,8. Growing evidence suggests that heterogeneous tumor cells can interact with TAMs to promote immune evasion and tumor progression. For example, in liver cancer, p53-inactivated cancer stem cells induce M2 macrophage polarization via the IL34-CD36 axis, facilitating immune escape 9. Glioblastoma stem cells secrete POSTN, which recruits TAMs by binding to integrin αvβ3, driving tumor progression 10. Tumor-initiating cells in pancreatic ductal adenocarcinoma induce TAMs to suppress CD8^+^ T cell driving antitumor response, leading to chemotherapy resistance 11. However, the mechanisms through which dormant TNBC tumor cells interact with TAMs to mediate ICB resistance remain unclear.

Currently, dormant tumor cells are primarily characterized by their reversible G0/G1 phase cell cycle arrest, positioning them as a potential root of tumor recurrence and metastasis and rendering them resistant to most clinical therapies 12,13. Therefore, understanding the biological functions of their characteristic genes is essential. In this study, we identified a significant upregulation of CCAAT/enhancer-binding protein beta (CEBPB) in dormant TNBC cells. CEBPB is a member of the CCAAT/enhancer-binding protein (C/EBP) family of transcription factors, and regulates biological processes such as cell proliferation, differentiation, immune responses, and tissue homeostasis 14-17. In addition to negatively regulating the cell cycle, CEBPB has garnered increasing attention for its role in tumor immunity 18,19. CEBPB expression in tumor cells has been shown to influence the expression of interferon response genes 20,21. Moreover, CEBPB can modulate the tumor microenvironment (TME) by regulating the expression of various secreted molecules, including cytokines and chemokines 22,23. Therefore, this study aims to elucidate the role of CEBPB in promoting tumor dormancy and modulating tumor immunity.

Here, we identified dormant tumor cells by utilizing a TNBC tumor model based on a label-retention system alongside a public scRNA-seq dataset from TNBC patients. Dormant tumor cells in TNBC exhibited resistance to anti-PD-1 therapy, characterized by high expression of CEBPB, and an immunosuppressive niche with reduced CD8^+^ T cells and increased CD206^+^ macrophages. We further found that elevated CEBPB expression induced tumor dormancy by transcriptionally activating genes such as CCNG2, which negatively regulate the cell cycle. Additionally, CEBPB promoted S100A8 expression to facilitate M2 macrophage polarization and immune evasion. Finally, paquinimod, a S100A8 inhibitor, exhibited a potential role in remodeling TME and improving ICB response. Overall, our results reveal a connection between tumor dormancy maintenance and immune evasion, and propos a therapeutic strategy for targeting dormant tumor cells to improve ICB efficacy in TNBC.

## Materials and Methods

### Cell lines and culture conditions

The HEK293T, MDA-MB-231 were purchased from Cell Bank of Chinese Academy of Science, Shanghai, China. Mouse TNBC cell line EMT6 was purchased form Xiamen Immocell Biotechnology Co., Ltd. Mouse TNBC cell line 4T07 was kindly provided by Fudan University Shanghai Cancer Center. Cells were cultured in Dulbecco's modified Eagle's medium (DMEM; Corning) supplemented with 10% fetal bovine serum (FBS) (Corning) and 1% Penicillin/ Streptomycin (M&C Gene Technology Ltd.) at 37 °C under a 5% CO_2_ atmosphere. All cell lines were identified by STR and regularly tested for mycoplasma contamination.

### siRNAs, plasmids, and lentiviral transfection

Two different siRNAs targeting different regions of *Cebpb* mRNA (siCEBPB-1 and siCEBPB-2), siRNA targeting *S100a8* mRNA (siS100A8) and negative control siRNA (siNC) were purchased from GenePharma (Shanghai). The siRNA sequences are detailed in **Table S1**.

TetOn-H2BeGFP plasmid (pSIN-TRE-H2BeGFP-hPGK-rtTA2) was a gift from Hector G. Palmer (Addgene plasmid, #165494). OVA expression plasmid (pLV3-CMV-OVAL(chicken)-3×FLAG-Fluc-hyg) was purchased form MiaoLing Plasmid Platform, China. Transient CEBPB over-expression plasmid pcDNA3.1-Cebpb (GenBank Accession Number: NM_017913.4) and pcDNA3.1-CEBPB (GenBank Accession Number: NM_001285879) were purchased form Fenghui Biotechnology, China. The CDS of *Cebpb* was optimized, synthesised and cloned into a pLenti vector. To create the knockdown plasmid of CEBPB and S100A8, we transcribed the sequences of siCEBPB-2, siS100A8 and siNC into shRNA format and cloned into pLKO vector separately. To create the conditional knockdown plasmid of CEBPB (CKD), we cloned the minimal U6-shCebpb cassette from pLKO-shCebpb plasmid, and replaced the minimal CMV-H2B-eGFP cassette of TetOn-H2BeGFP plasmid. We also added a T2A-NeoR cassette after rtTA2 cassette for selection.

To create the firefly luciferase reporter of S100A8 transcriptional activity, we cloned a 529 bp fragment of *S100a8* promoter region from mouse gDNA into pGL3-basic plasmid. We synthesized a double-site mutation fragment and replaced both CEBPB binding sites to create the binding site mutation plasmid. The pRL-SV40 plasmid encoding renilla luciferase was kept by our laboratory. All constructions were conducted with In-Fusion Snap Assembly (Takara, #638947).

Cell transfection was carried out by using Lipofectamine 3000 Transfection Reagent (Invitrogen, #11668019) according to the manufacturer's instructions. For lentiviral transfection, HEK293T cells were transfected with psPAX2, pMD2.G and transfer plasmid (TetOn-H2BeGFP vector, overexpression vector, shRNA vector or CKD vector). Viral supernatants were collected twice at 48 and 72 h after transfection. Target cells were transduced with viral supernatants in the presence of 5 μg/mL polybrene (Merck Millipore). After 24 hours, stable infected cells were selected with corresponding concentrations of puromycin (Selleck Chemicals, #S7417), G418 (Selleck Chemicals, #S3028) or hygromycin B (Selleck Chemicals, #S2908) for at least one week.

### Animal experiments and treatments

Female BALB/c or BALB/c nude mice, aged 4-6 weeks, were purchased from GemPharmatech Co., Ltd. A suspension of either 1×10^6^ 4T07 or 5×10^5^ EMT6 tumor cells in 100 μL PBS was injected into the fourth pair of mammary fat pad of each mouse. For dormant tumor cell chasing, mice received 2 mg/mL doxycycline (DOX, Solarbio, #D8960) in drinking water ad libitum until the tumors reached approximately 100 mm³.

For drug therapy treatments, once tumors reached approximately 100 mm³, mice with similar tumor burdens were randomized into treatment groups as described below. In the CEBPB-CKD tumor experiment, mice were treated with 2 mg/mL DOX ad libitum in drinking water. For the anti-PD-1 treatment experiment, mice received an intraperitoneal injection of 200 μg anti-PD-1 antibody (Junshi Biosciences) or IgG control every 3 days. In the paquinimod treatment experiment, mice were administered 10 mg/kg paquinimod (Selleck, #S9963) or control vehicle via oral gavage every 2 days.

Tumor volume and body weight were measured twice weekly, with tumor volume calculated as 0.5 × width^2^ × length. At the experimental endpoint, mice were euthanized, tumors were removed and weighed, and further analyses were conducted. The experimental endpoint for each mouse was defined as either a tumor volume >2000 mm³ or a noticeable difference in tumor volume between control and experimental groups.

To assess survival time, a separate group of mice underwent the same experimental treatment, with tumor volume monitored every 2-3 days. When tumor volume in any group reached 2000 mm³, mice were euthanized, marking the experimental endpoint, and recorded as deceased. Survival time was calculated from the day of tumor inoculation.

### Immunofluorescence and immunohistochemistry staining

For immunofluorescent staining, tumor tissues were harvested and embedded with OCT compound (Sakura) at -80 °C overnight. Frozen sections (8 µm) were fixed with 4% paraformaldehyde (PFA) in PBS, and then permeabilized with acetone. The sections were subsequently blocked with blocking solution for 1 hour at room temperature (RT). Primary antibodies were applied at 4 °C overnight: active-Caspase-3 (1:100, #9665, Cell Signaling), Ki67 (1:100, #ab15580, Abcam), CD8a (1:100, #ab217344, Abcam), F4/80 (1:100, #ab300421, Abcam), F4/80 (1:100, Cat #123116, Biolegend), CD206 (1:100, #ab300621, Abcam), MHC Class II (I-A/I-E) (1:100, #14-5321-82, Invitrogen), CEBPB (1:100, #PA5-79030, Invitrogen). Secondary antibodies (Alexa Fluor™ 555 (1:500, #A-21428, Invitrogen), Alexa Fluor™ Plus 647 (1:200, #A32733, Invitrogen), Cy3 (1:200, #P0153, Beyotime)) and Hoechst 33342 (1:1000, #62249, Thermo Scientific) were then added for 1 hour at RT. Images were captured using a Leica-DMi8 invert microscope and analyzed with ImageJ Software.

For immunohistochemistry staining, tumor tissues were fixed with 4% PFA in PBS overnight at RT and embedded in paraffin. Formalin-fixed, paraffin-embedded (FFPE) sections (5 µm) were deparaffinated, rehydrated and treated with either 1mM EDTA buffer or 10 mM sodium citrate buffer dependent on the primary antibody requirements. Following antigen retrieval, sections were blocked with 3% hydrogen peroxide and 3% bovine serum albumin. Primary antibodies (CD8a (1:1000, #ab217344, Abcam), F4/80 (1:1000, #ab300421, Abcam), CD206 (1:1000, #ab300621, Abcam)) were incubated overnight at 4 °C. The sections were then treated with HRP-conjugated goat anti-rabbit antibody, followed by diaminobenzidine (DAB) and hydrogen peroxide for detection and visualization. Images were captured using a NanoZoomer S360 scanner, and analyzed with ImageJ software.

### Tissue microarray and multiplex immunohistochemistry staining

A tissue microarray (#TNBC-1601) containing 80 paired TNBC samples and adjacent normal tissues was obtained from Shanghai SuperBiotek CO., LTD. Immunohistochemistry staining for CEBPB was performed using a specific anti-CEBPB antibody (1:500, #PA5-79030, Invitrogen). The proportion of positively stained cells was categorized as 0 (<5%), 1, (5-25%), 2 (25-50%), 3 (50-75%), 4 (75-100%). Staining intensity was graded on a scale from 0 to 3, where 0 was negative, 1 was weak, 2 was moderate, 3 was strong. The total score of CEBPB staining was determined was calculated by multiplying proportion scores by intensity scores. The approval for research using human tissue samples obtained from the Medical Ethics Committees of Shanghai East Hospital, Tongji University.

Multiplex immunohistochemistry staining was conducted on the tissue microarray. Following standard deparaffinization, rehydration, and treatment with 1 mM EDTA buffer, slides were blocked and incubated with primary antibodies for 1 hour at RT. The primary antibodies used were: CEBPB (1:200, #PA5-79030, Invitrogen), CD68 (1:500, #ab201340, Abcam), CD163 (1:800, #24595, Cell Signaling), CD8a (1:250, #ab237709, Abcam); or CEBPB, Ki67(1:200, #ab15580, Abcam), and S100A8(1:1000, #ab92331, Abcam). HRP-labeled polymer secondary antibodies were then applied for 30 minutes at RT, followed by a 10 minute incubation with corresponding fluorophore-conjugated TSA. The TSA used were: iFluor 594 tyramide (1:600, #45107, ATTbio), iFluor 488 tyramide (1:400, #11060, ATTbio), Cy5 tyramide (1:400, #11066, ATTbio) and iFluor 430 tyramide (1:400, #45096, ATTbio), or iFluor 594 tyramide (1:600, #45107, ATTbio), Cy5 tyramide (1:400, #11066, ATTbio) and iFluor 488 tyramide (1:400, #11060, ATTbio). DAPI was applied to stain nuclei. All slides were scanned using an Pannoramic SCAN II slide scanner (3DHISTECH., Ltd).

### Flow cytometry and fluorescence activated cell sorting

Tumor tissues were digested into single cells by 1mg/mL collagenase IV (Yeasen, #40510ES76) and 20mg/mL DNase I (Yeasen, #10325ES80) for 15 minutes at 37 ℃. The resulting cell suspensions were filtered through a 100 µm cell strainer and washed with PBS three times. Red blood cells were removed using an RBC lysis buffer (Solarbio, #R1010). Fixable Viability Dye eFluor™ 780 (eBioscience, #65-0865-14) or DAPI (Beyotime, #C1005) were then used to exclude dead cells. Cells were incubated with cell surface-specific antibodies at RT for 20 minutes in the dark. For intracellular staining, samples were fixed using IC fixation buffer (eBioscience, #FB001) for 20 minutes, followed by twice washes with 1X permeabilization buffer (eBioscience, #00-8333-56). Samples were then incubated with antibodies targeting intracellular antigens in 1X permeabilization buffer for 20 minutes. The following fluorescent dye-conjugated antibodies were used for staining: CD45-BV421 (Biolegend, #103134), CD8a-APC (Biolegend, #155005), CD11b-PE (Biolegend, #101207), F4/80-APC (Biolegend, #123116), F4/80-PC5.5 (Biolegend, #123127), PD-1-PC7 (Biolegend, #135215), CD206-APC (eBioscience, #17-2061-82), CD206-PC7 (eBioscience, #25-2061-82), MHC Class II (I-A/I-E)-FITC (eBioscience, #11-5321-81), Arg1-PC7 (Invitrogen, #25-3697-80), iNOS-FITC (Invitrogen, #53-5920-80), CD206-APC (Invitrogen, #17-2069-41), TNFa-FITC (Invitrogen, #11-7321-81), IL12/23-PerCP-Cy5.5 (Invitrogen, #45-7123-82). Afterwards, samples were conducted using a CytoFLEX flow cytometer (Beckman, **Figure S3B**) and cell sorting was performed using the Aria III cell sorter (BD Biosciences). For dormant tumor cell sorting, the top 1% of H2BeGFP-retaining cells were sorted as eGFP^+/high^ dormant cancer cells, while cells with moderate fluorescence intensity were classified as eGFP^-/low^ proliferating cells. Data were analyzed with FlowJo software.

### Preparation of conditional medium

4T07, EMT6 and MDA-MB-231 cells were transfected with pcDNA plasmids for CEBPB overpression, or siRNAs for CEBPB knockdown. Cells were cultured in FBS-free medium for 3 days to harvest conditional medium. The supernatants were collected and filtered using a 22 µm cell strainer. The supernatants were further concentrated approximately ten-fold using an ultrafiltration tube with a 3 kDa molecular weight cutoff (Millipore, #UFC9003) by centrifugation at 12,000 rpm. The protein content in conditional medium was quantified by BCA assay, and aliquots were stored at -80 ℃.

### Preparation of bone marrow derived macrophages (BMDMs)

Bone marrow (BM) cells were acquired from the femurs and tibias of 6 to 8 week-old BALB/c mice. The BM cells were filtered through a 100 µm cell strainer, and red blood cells were removed using an RBC lysis buffer (eBioscience, #00-4300). BM cells were then differentiated in complete DMEM medium supplemented with 20 ng/mL recombined mouse M-CSF protein (MCE, #HY-P70263A) for 7 days. The medium was changed every 3 days with fresh complete DMEM medium containing M-CSF. Flow cytometry confirmed that over 90% cells were F4/80^+^CD11b^+^ macrophages.

### Preparation of human peripheral blood mononuclear cell-derived macrophages (PBMC-Mφ)

Human blood monocytes were isolated from healthy donor. Peripheral blood mononuclear cells (PBMCs) were isolated using a Ficoll-Paque PLUS (Cytiva, #17144002) density gradient, and subsequently monocytes were isolated from PBMCs using anti-CD14 magnetic beads (Biolegdend, #480093) according to the manufacturer's protocol. Purified CD14^+^ PBMC cells were then differentiated in complete RPMI-1640 medium supplemented with 1% GlutaMax, 50 mM β-mercaptoethanol and 50 ng/mL recombined human M-CSF protein (MCE, #HY-P73827) for 7 days. The medium was changed every 3 days with fresh complete RPMI-1640 medium containing M-CSF. Flow cytometry confirmed that over 90% cells were CD68^+^ macrophages.

### Cell migration assay

A total of 1 × 10^5^ cells in 300 μL FBS-free DMEM medium were seeded into the upper chamber of a 24-well Boyden chamber (Corning, #3422). The lower chamber was filled with 600 μL of complete DMEM or conditioned medium supplemented with 10% FBS and 1% penicillin-streptomycin. After 24 hours of incubation, non-migratory cells on the top membrane of the upper chamber were removed, and migratory cells on the bottom membrane were stained with 1% crystal violet for 30 minutes and washed twice with PBS. Images were acquired using the ZEISS AX10 system.

### Macrophage polarization assay

BMDMs or PBMC-M**φ** cells were co-cultured in conditional medium supplemented with 10% FBS and 1% penicillin-streptomycin, or in complete DMEM or 1640 medium supplemented with rS100A8 (MCE, #HY-P73671 for BMDMs and #HY-P70531 for PBMC-M**φ**) or rCXCL1 (MCE, #HY-P7188 for BMDMs) for 48 hours. After incubation, cells were collected, and their polarization was assessed by flow cytometry.

### T cell isolation assay

CD8^+^ T cells were isolated from spleens of 6-8-week-old BALB/c mice or OT-1 mice using the Naive CD8a^+^ T Cell Isolation Kit (Miltenyi Biotec, #130-096-543), following the manufacturer's instructions. Fresh spleens were harvested and smashed with a plunger in cold PBS. Cells were filtered through a 100 µm cell strainer and red blood cells were removed by RBC lysis buffer at RT. A total of 1 × 10^7^ cells were stained with 10 µl naive CD8a^+^ T cell Biotin-antibody cocktail at 4 ℃ for 5 minutes, and then stained with 20 µl anti-Biotin MicroBeads and 10 µl CD44 MicroBeads for another 10 minutes. Cells were washed with isolation buffer, and loaded to a LS column and separated in a magnetic field.

### T cell proliferation assay

The isolated CD8^+^ T cells were cultured on 0.5 mg/mL anti-mouse CD3e (Bioxcell, #BE0261, Clone KT3) and 5 mg/mL anti-mouse CD28 (Bioxcell, #BE0328, Clone D665) pre-coated plates with T cell culture medium (complete DMEM supplemented with 1% GlutaMax, 1% sodium pyruvate, 1% NEAA, 1% HEPES, 50mM β-mercaptoethanol and 240 U/mL IL-2 (MCE, #HY-P7077). After 48 hours of activation, the CD8^+^ T cells were labeled with CellTrace™ Violet dye (Invitrogen, #C34571) and co-cultured with BMDMs at a 1:1 ratio for 48h.

### T cell or IFN-γ mediated killing assay

For IFN-γ cytotoxic assay, EMT6-TetOn tumor cells were pulsed with 5 μg/mL DOX and chased for 10 days. Tumor cells were treated with IFN-γ (MCE, #HY-P7071) at concentrations of 0, 0.1, 0.5, 1, 5, 10, and 20 ng/mL. After 48 hours, tumor cells from each well were collected, and the proportion of eGFP^+/high^ cells in tumor cells was analyzed by flow cytometry.

For T cell killing assay, CD8a^+^ T cells were extracted from OT-1 mice. EMT6-TetOn cells were stabled trastected with OVA overexpression vector, and performed the same chasing steps as outlined in the previous paragraph. T cells were co-cultured with tumor cells at ratios of 0:1, 2.5:1, 5:1 and 10:1 in T cell culture medium. After 48 hours, tumor cells from each well were collected, and the proportion of eGFP^+/high^ cells in tumor cells was analyzed by flow cytometry.

### Total RNA isolation and qPCR analysis

Total RNA from the indicated cell lines was isolated with Trizol reagent (Sigma Aldrich, #T9424) and then used to synthesize cDNA using the PrimeScript™ RT reagent kit (Takara, #RR047A). qPCR analysis was performed using TB Green Premix Ex Taq II (Takara, #RR420A) on an ABI QuantStudioTM 6 Flex system (Thermo Fisher Scientific) following the manufacturer's instructions. GAPDH served as the internal control, and relative mRNA expression levels were quantified using the 2-ΔΔCt method. The qPCR primer sequences are detailed in **Table S1**.

### Western blot

Whole cell lysates were collected from 4T07 and EMT6 cells using RIPA lysis buffer supplemented with a protease inhibitor. Protein concentration was measured with BCA assay, and 20 μg of each sample was loaded onto SDS-PAGE, separated using a 5% spacer gel and a 12% separating gel, and transferred to a PVDF membrane. After blocking in 5% milk in PBST, the membranes were incubated overnight at 4°C with primary antibodies: CEBPB (1:1000, #ab32358, Abcam), CCNG2 (1:1000, #AV03032, Sigma Aldrich), p27 (1:1000, #ab193379, Abcam), and S100A8 (1:1000, #47310T, Cell Signaling). Following three washes with PBST, the membranes were incubated with secondary antibodies for 1 hour at RT. Images were acquired using an Odyssey system (Li-COR).

### Tumor cell proliferation assay

A Cell Counting Kit-8 assay was used to assess the growth ability of tumor cells. Briefly, 4T07 or EMT6 cells were seeded in a 96-well plate at densities of 1000 or 500 cells per well, respectively. Then, 10 μL of Cell Counting Kit-8 regent (Dojindo Laboratories, #CK04) was added to each well. After 75 min of incubation at RT, cellular absorbance was measured at 450 nm using the SpectraMax M5 Microplate Reader (Molecular Devices).

### Colony formation assay

In the colony formation assay, 4T07 or EMT6 cells were plated in 6-well plates at a density of 1000 cells per well and incubated for approximately 2-3 weeks. The resulting colonies were then fixed with 4% PFA and stained with 0.5% crystal violet. The average number of colonies was calculated using ImageJ software.

### Cell cycle assay

Cells were fixed in 70% cold ethanol at 4 °C overnight. After fixation, cells were incubated with 500 μL PI/RNAse staining solution (Invitrogen, #F10797) for 30 min in the dark. Cell cycle analysis was performed using a CytoFLEX flow cytometer (Beckman) and evaluated with FlowJo software.

### Chromatin immunoprecipitation assay (ChIP) and ChIP-seq

ChIP assay was conducted following the manufacturer's instructions of EZ-Magna ChIP®A/G (Sigma-Aldrich, #17-10086). Cells were fixed with 37% formaldehyde for 10 minutes and neutralized with 10X glycine solution for 5 minutes at RT. Cells were collected in SDS solution and chromatin shearing were conducted by ultrasonication for 15 minutes. 25 µg of sheared chromatin was utilized for each IP reaction. 4 µg CEBPB antibody (Abcam, #ab32358) was used for CEBPB IP, equivalent IgG antibody (Cell Signaling, # 3900) was used as IP control, and mock IP was also conducted as Input control. A protein A/G beads (Invitrogen, #80106G) was used to bind the antibody-chromatin-DNA complex and incubated with rotating at 4 ℃ overnight. DNA was then separated from the complex and purified, making it suitable for downstream qPCR and DNA electrophoresis analysis. Enrichment of chromatin regions was determined by the ΔCt method and normalized with Input control. The qPCR primer sequences are detailed in **Table S1**.

For ChIP-seq, the DNA purity of input and IP- CEBPB in 4T07 cells was checked using the NanoPhotometer® spectrophotometer (IMPLEN). A sequencing library was then constructed by Novogene Corporation. Paired-end sequencing (150 bp) was subsequently performed on Illumina platform (Illumina). Reads were aligned to the Mus musculus genome (GRCm39) using BWA. Peaks in individual samples were identified with MACS2, applying a q-value threshold of 0.05. Finally, ChIPseeker was employed to annotate the genomic regions corresponding to each peak (**Table S5**).

### Dual-luciferase reporter assay

HEK293T Cells were seeded in a 24-well plate. 250 ng pcDNA3.1-Blank or pcDNA3.1-Cebpb, 250 ng pGL3- S100a8 pro-WT or pGL3- S100a8 pro-Mut and 2.5 ng pRL-SV40 were co-transfected by Lipofectamine 3000 and cultured for 72 hours. Firefly luciferase activity and Renilla luciferase activity were measured with Glo/Max luminometer in accordance with the manufacturer's protocols (Promega, #E1910). Relative luciferase activity was calculated as the ration of Firefly luciferase luminous intensity to Renilla luciferase luminous intensity (Luc/RLuc) and normalized with control group.

### RNA-seq analysis

Total RNA was isolated from OE-CEBPB and OE-Control 4T07 cells using TRIzol reagent (Sigma Aldrich, #T9424). The RNA integrity was then assessed with the RNA Nano 6000 Assay Kit on a Bioanalyzer 2100 system (Agilent Technologies). Next, cDNA libraries were prepared through Poly-A sequencing and sequenced on an Illumina NovaSeq platform with 150 bp paired-end reads. Clean reads were subsequently aligned to the Mus musculus genome (GRCm39) using HISAT2. The raw counts of reads per gene per sample were obtained using featureCounts. Differential gene expression between experimental groups was assessed with the DESeq2 package in R. Genes with raw counts of less than 10 per sample were excluded from analysis. Differential expressed genes (DEGs) were identified with absolute log2 fold change greater than 0 and adjusted p-value less than 0.05 (**Table S4**).

### Single-cell RNA-seq analysis

Single-cell RNA sequencing data for TNBC patients were obtained from Ayse Bassez *et al.*, who analyzed changes in the tumor microenvironment before and after neoadjuvant anti-PD-1 therapy in 29 breast cancer patients 24. Read counts for each patient were accessed publicly via biokey.lambrechtslab.org. Data normalization, dimensionality reduction, and clustering were performed from 12 TNBC patients using the SeuratV4 package in R. Cell types were annotated based on cell-specific marker genes from the original reference study. Cells identified as "tumor cells" were extracted and subjected to further unsupervised clustering. DEGs were identified based on the FindMarkers function in Seurat with absolute log2 fold change greater than 0.5 and adjusted p-value less than 0.05. Cell cycle analysis was conducted using Seurat's built-in cell cycle scoring function, labeling each tumor cell according to its cell cycle phase score (G1, S, or M). Gene Set Variation Analysis was conducted on the average expression of cells in each group using the GSVA package in R. In addition, the percentage of a cell subpopulation refers to its proportion out of the patient's total cells or main cell type count.

To identify dormant tumor cells in patients, we performed signature scoring analysis on dormant tumor cell related signatures. The "Signature_LRC_Dormant" signature comprised the top 300 up-regulated DEGs in dormant tumor cells identified via a label-retention method in the MDA-MB-231 cell line (GSE267757). Additionally, the “Signature_PanCa_Dormant” signature included the top 300 up-regulated DEGs in dormant tumor cells identified by label-retention in melanoma, colorectal cancer, and glioblastoma cell lines from the team of Héctor G. Palmer 25. Using the AddModuleScore function in Seurat, we integrated these dormant tumor cell related signatures into the target object, enabling each cell to receive a score for each signature. We then compared differences in signature scores across cell subgroups at the single-cell level.

Signature scoring analysis on dormant tumor cell related signatures also performed using RNA-seq from mouse mammary tumors treated with a combination of anti-PD-1 and anti-CTLA4 antibodies for 7 days with 18 responders and 4 non-responders (GSE124821); RNA-seq data of 53 advanced melanoma patient pretreated samples (nivolumab) from the CA209-038 study with 3 responders and 46 non-responders 26. Clinical assessment of CEBPB expression was performed from TCGA-BRCA (transcriptomic-level data) and CPTAC-BRCA (protein-level data) datasets with breast cancer subtype annotations.

### Statistical analysis

Venn plot was visualized via Venn Diagram R package. Violin plots and Bar plots were generated by ggplot2 R package. For the Kaplan-Meier survival analysis, log-rank tests were applied via GraphPad Prism 9 (GraphPad Software) or R version 4.2.1 (www.r-project.org). The data are presented as mean ± standard deviation and analyzed using GraphPad Prism 9 or R version 4.2.1. Two unpaired groups were compared using a two-tailed unpaired Student t test. Statistical significance is reported as follows: n.s., not significant, *, *P* < 0.05, **, *P* < 0.01, ***, *P* < 0.001.

## Results

### Dormant tumor cells reside an immunosuppressive niche and exhibit resistance to ICB in TNBC

**Figure 1:**
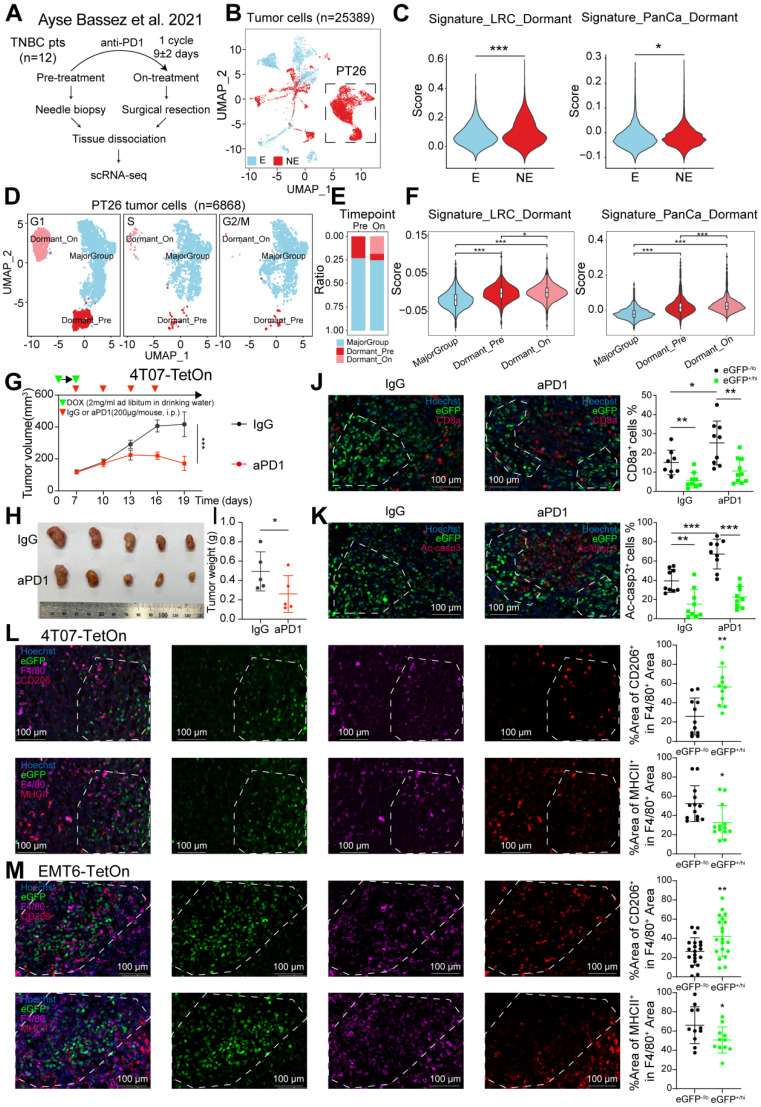
** Role of dormant tumor cells in residing an immunosuppressive niche and mediating ICB resistance. A-B**, Umap plot of 25389 tumor cells from a cohort of 12 TNBC patients in the scRNA-seq dataset published by Ayse Bassez *et al.* 24, categorized into clonotype expansion (E, blue) and no/limited expansion (NE, red) groups based on T cell clone expansion following anti-PD-1 therapy. **C**, Violin plots showing expression scores of Signature_LRC_Dormant and Signature_PanCa_Dormant in tumor cells from a cohort of 12 TNBC patients. **D-E**, Umap plots and stacked histogram of cell cycle score analysis categorizing 6868 tumor cells from the PT26 patient into Dormant_Pre, Dormant_On, and Major_group clusters. **F**, Violin plots showing expression scores of Signature_LRC_Dormant and Signature_PanCa_Dormant in tumor cells from the PT26 patient. **G-I**, Tumor volume (G), image (H) and weight (I) of 4T07-TetOn allografs in BALB/c mice (*n* = 5). Tumor-bearing mice were randomly divided into IgG or anti-PD-1 (aPD1) treatment group until tumor volume reached approximately 100 mm^3^. **J-K**, Representative immunofluorescene (IF) images of CD8a (J) and active-Caspase3 (K) staining in two treatment groups of serial tumor sections. White dashed line marks eGFP^+/high^ area. **L-M**, Representative IF images of F4/80 and CD206 or F4/80 and MHCII staining in 4T07-TetOn (L) or EMT6-TetOn (M) serial tumor sections. White dashed line marks eGFP^+/high^ area. *, *P* < 0.05, **, *P* < 0.01, ***, *P* < 0.001.

Dormant tumor cells are a heterogeneous subgroup within tumors. Recent advances in single-cell RNA sequencing (scRNA-seq) have accelerated research on tumor heterogeneity 28,29. Ayse Bassez's team analyzed intra-tumoral microenvironment changes before and after neoadjuvant anti-PD-1 therapy in breast cancer patients using scRNA-seq (**Figure 1A**). We reanalyzed this scRNA-seq data from a cohort of 12 TNBC patients, categorizing tumor cells into clonotype expansion (E) and no/limited expansion (NE) groups based on T cell clone expansion post anti-PD-1 therapy 24 (**Figure 1B**). Using the TNBC dormant tumor cell signature of GSE267757 (Signature_LRC_Dormant) and the pan-cancer dormant tumor cell signature (Signature_PanCa_Dormant) from Héctor G. Palmer's team 25, we found that both signatures had higher scores in the NE group compared to the E group (p < 0.05) (**Figure 1C**). There were also significantly elevated scores of these two signatures in the ICB non-responsive group compared to the responsive group in two additional public datasets of ICB-treated tumors (**Figure S1A-B**). Then, cell cycle score analysis classified these tumor cells into distinct clusters: a G1-phase-dominant cell cluster (Dormant) and a major population (MajorGroup) (**Figure S1C and Table S2**). Notably, the scores of two signatures were significantly elevated in Dormant compared to MajorGroup, with the highest scores observed specifically in the post-treatment G1-phase-dominant cell cluster (Dormant_On) (**Figure S1D**). Surprisingly, patient No. 26 (PT26) in the NE group had the most abundance of tumor cells, which caught our attention (**Figure 1B**). Further analysis revealed three distinct clusters in PT26's tumor cells: Dormant_Pre, Dormant_On, and MajorGroup (**Figure 1D-E and Figure S1E**). Tumor cells in the first two clusters showed enhanced scores of dormant tumor cell signatures (**Figure 1F and Table S3**. This comprehensive analysis suggested that dormant tumor cells exist in TNBC patients and may enrich in tumors with limited T cell expansion.

To identify and enrich dormant tumor cells from bulk tumor cells in TNBC, we adopted a TetOn-H2BeGFP label system, which enables convenient tracking of cells in both *in vivo* and *in vitro* experiments 25,30-34. This system utilizes a tetracycline-responsive element to regulate the expression of H2B-eGFP fusion protein in a lentiviral vector 25 (**Figure S1F**). We stably transfected the murine TNBC cell lines 4T07 and EMT6 with this vector, creating 4T07-TetOn and EMT6-TetOn cells. *In vitro*, cells uniformly expressed nuclear eGFP following doxycycline (DOX) treatment, and eGFP fluorescence gradually diluted with cell division after DOX withdrawal. Dormant tumor cells, due to their slow cycling nature, retained higher levels of eGFP fluorescence after 12 days of chasing (**Figure S1F**). In allografts established from 4T07-TetOn or EMT6-TetOn cells, eGFP fluorescence was observed at all chasing time points, with high intensity in most tumor cells on day 0, which gradually decreased over time. After 12 days of chasing, niches enriched in dormant tumor cells had formed (**Figure S1G**). Thus, we identified dormant TNBC cells both *in vitro* and *in vivo*.

To explore whether dormant TNBC cells could survive from immunotherapy, we conducted an *in vivo* anti-PD-1 therapy assay. 4T07-TetOn cells were orthotopically transplanted into the fourth pair of mammary fat pads in female BALB/c mice. Anti-PD-1 or IgG treatment was administered after tumor masses reached 100 mm³, with DOX withdrawal (**Figure 1G**). We observed that anti-PD-1 therapy partially inhibited tumor growth (**Figure 1H-I**). Immunofluorescence (IF) analysis revealed a significant increase in CD8^+^ T cell infiltration in the anti-PD-1 group compared to the control group (**Figure 1J**). Further analysis showed large active-caspase-3-positive area in the tumor centers of both groups, indicating extensive apoptosis. Dormant tumor cells (GFP^+/hi^) clustered around these apoptotic regions. Interestingly, rapidly proliferating tumor cells (GFP^-/lo^) exhibited increased apoptosis in the anti-PD-1 group compared to the IgG group (**Figure 1K**). Meanwhile, dormant tumor cells formed clusters were mutually exclusive with active-caspase-3 in both groups, suggesting that dormant tumor cells can evade CD8^+^ T cell- mediated killing.

Both our study and others have demonstrated that dormant tumor cells are intrinsically resistant to T cell-mediated killing (**Figure S1H-J**). Here, we primarily focus on the potential immunosuppressive mechanisms that involve the interactions between dormant tumor cells and immune cells within the TME. TAMs have been observed to interact with cancer stem cells and tumor-initiating cells 9-11. Our previous research also identified a strong correlation between dormant tumor cells and TAMs in TNBC 7. Accordingly, IF analysis revealed that dormant tumor cell niches were frequently infiltrated by macrophages, predominantly CD206-expressing M2 macrophages, with fewer MHCII-expressing M1 macrophages in both 4T07-TetOn and EMT6-TetOn allografts (**Figure 1L-M and Figure S1K**). The aforementioned scRNA-seq analysis further confirmed a reduction in the proportion of T cells in the NE group. Notably, the NE group displayed marked enrichment of C7_CX3CR1 macrophages with M2-like phenotype, while C3_CCR2 macrophages expressing M1 markers were significantly reduced (**Figure S1L-M**). TAMs, predominantly M2 macrophages, are recognized as important contributors to immunosuppression 35. Therefore, dormant TNBC cells may occupy a TAM-enriched niche for immune evasion.

### CEBPB as a key factor of dormant tumor cells in TNBC

Several studies have explored the characteristics of tumor dormancy in TNBC at the transcriptomic level. The GSE267757 dataset used various label-retaining methods to track long-term dormant tumor cells in human TNBC cell line MDA-MB-231. Pilar Baldominos' team utilized the mVenus-p27K^-^ reporter to identify the transiently dormant tumor cells in a murine TNBC cell line D2A1 derived allograft (GSE198713). To identify representative biomarkers of dormant tumor cells in TNBC, we conducted a differential expressed gene analysis and identified nine genes that were significantly upregulated in both datasets. Among these genes, CEBPB, a member of the C/EBP family that has been reported previously to induce cell cycle arrest or senescence 36-39, caught our attention (**Figure S2A-C**).

**Figure 2:**
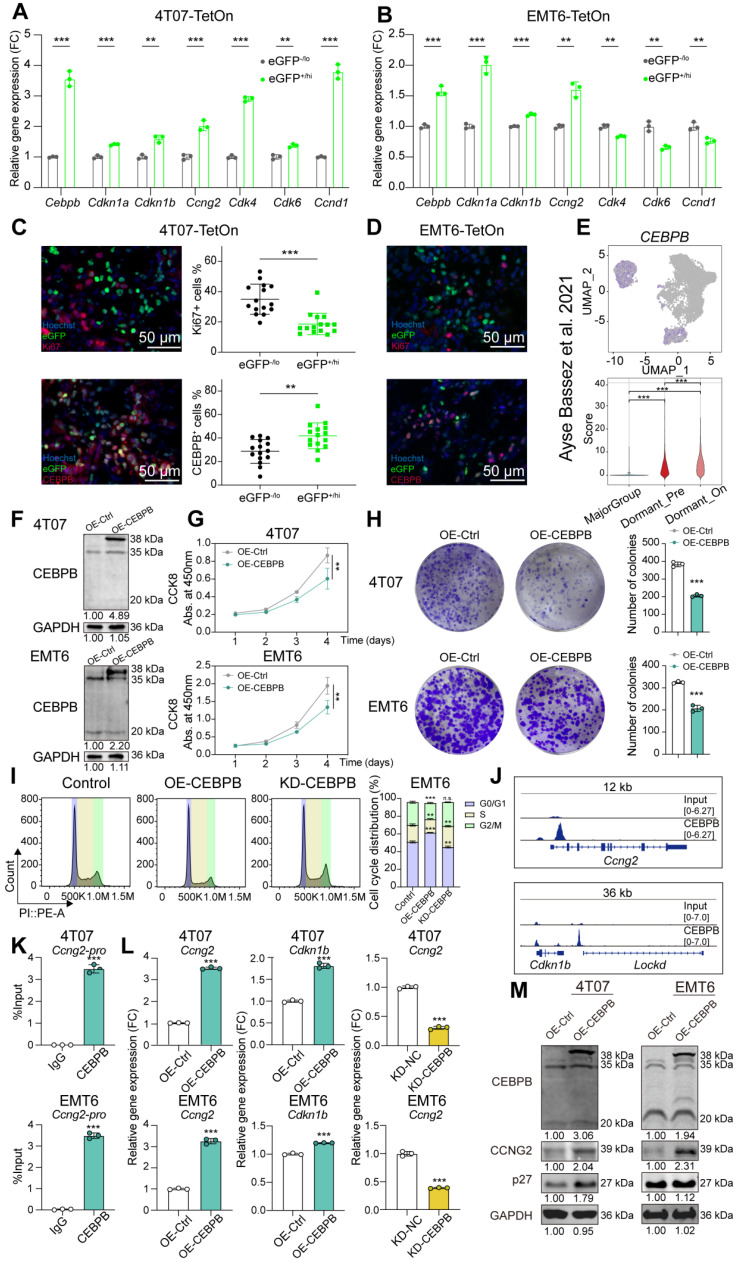
** CEBPB as an essential factor in TNBC tumor dormancy. A-B**, mRNA expression of *Cebpb* and tumor dormancy related cell cycle markers in eGFP^-/lo^ and eGFP^+/hi^ tumor cells sorted from 4T07-TetOn (A) or EMT6-TetOn (B) tumor masses.** C-D**, Representative IF images of Ki67 and CEBPB staining in 4T07-TetOn (C) or EMT6-TetOn (D) serial tumor sections.** E**, Expression score of *CEBPB* in tumor cells from PT26 patient in the scRNA-seq data published by Ayse Bassez *et al.* 24.** F**, Overexpression of CEBPB in 4T07 and EMT6 cell lines were detected by western blot. **G-H**, Cell viability assessed by Cell Counting Kit-8 assay (G) and colony formation assay (H) in 4T07 and EMT6 cells with or without CEBPB overexpression. **I**, Cell cycle analysis of EMT6 cells with CEBPB overexpression or knockdown. **J**, CEBPB characteristic binding peaks for *Ccng2* and *Cdkn1b* from anti-CEBPB ChIP-seq in 4T07 cells. **K**, ChIP-qPCR showing the CEBPB binding region in the *Ccng2* promoter of 4T07 and EMT6 cells. **L**, mRNA expression of *Ccng2* or *Cdkn1b* in 4T07 and EMT6 cells with CEBPB overexpression or knockdown. **M**, Protein expression of CCNG2 or p27 in 4T07 and EMT6 cells with CEBPB overexpression or knockdown. **, *P* < 0.01, ***, *P* < 0.001.

We then confirmed the potential role of CEBPB in TNBC tumor dormancy. Allografts derived from 4T07-TetOn and EMT6-TetOn cells were pulsed with DOX for 7 days and chased for another 12 days. After chasing, the tumors were excised and dissociated into single cells. Fluorescence activated cell sorting of dormant tumor cells showed higher mRNA levels of cell cycle inhibitors *Cdkn1a*, *Cdkn1b* and *Ccng2* compared to rapidly proliferating tumor cells, reinforcing their G0/G1 cell cycle arrest nature (**Figure 2A-B**). Dormant tumor cells also exhibited higher expression levels of *Cebpb*, consistent with our screening results (**Figure 2A-B**). IF staining further revealed that dormant tumor cells had no significant colocalization with Ki67 protein, but strongly co-localized with CEBPB protein in tumor sections (**Figure 2C-D**). In addition, scRNA-seq data demonstrated that *CEBPB* was significantly upregulated in dormant-like cells (Dormant_Pre and Dormant_On) compared to the major group (**Figure 2E**). Thus, we confirmed that CEBPB is highly expressed in dormant TNBC cells across cell lines, mouse allografts, and patient tumors.

We further investigated how CEBPB regulates TNBC tumor dormancy. We initially analyzed CEBPB mRNA and protein expression levels across different breast cancer subtypes from TCGA-BRCA and CPTAC-BRCA, separately. And we found that CEBPB expression is elevated in the TNBC subtype (**Figure S2D**). We then performed immunohistochemical staining for CEBPB on a tissue microarray (TMA) comprising 80 pairs of tumor and adjacent normal tissues from TNBC patients. The results demonstrated significantly higher CEBPB expression in TNBC tumor tissues compared to adjacent normal tissues (**Figure S2E**). In the survival analysis, patients in the high-expression group (median expression as cutoff) exhibited a slightly shorter overall survival, although there was no significant difference (**Figure S2F**). We then modulated its expression levels (**Figure 2F and Figure S2G-I**). Overexpression of CEBPB in 4T07 and EMT6 cell lines significantly inhibited cell growth and proliferation *in vitro* and in allografts derived from nude mice, while knockdown of CEBPB significantly promoted cell proliferation (**Figure 2G-H and Figure S2J-M**). Cell cycle analysis showed that CEBPB induced G1-phase cell cycle arrest in tumor cells (**Figure 2I**). ChIP-seq analysis revealed robust binding of CEBPB in the promoter region of *Ccng2* and remote enhancer region of *Cdkn1b*, which was further validated by ChIP-qPCR (**Figure 2J-K and Figure S2N-P**). qPCR and western blot analyses confirmed that CEBPB promoted the expression of these genes (**Figure 2L-M and Figure S2Q**). Collectively, CEBPB-high dormant tumor cells undergo G1-phase cell cycle arrest by activating CCNG2 and p27 in TNBC.

### Highly expressed CEBPB in TNBC cells drives M2 macrophage polarization, leading to immune evasion

**Figure 3:**
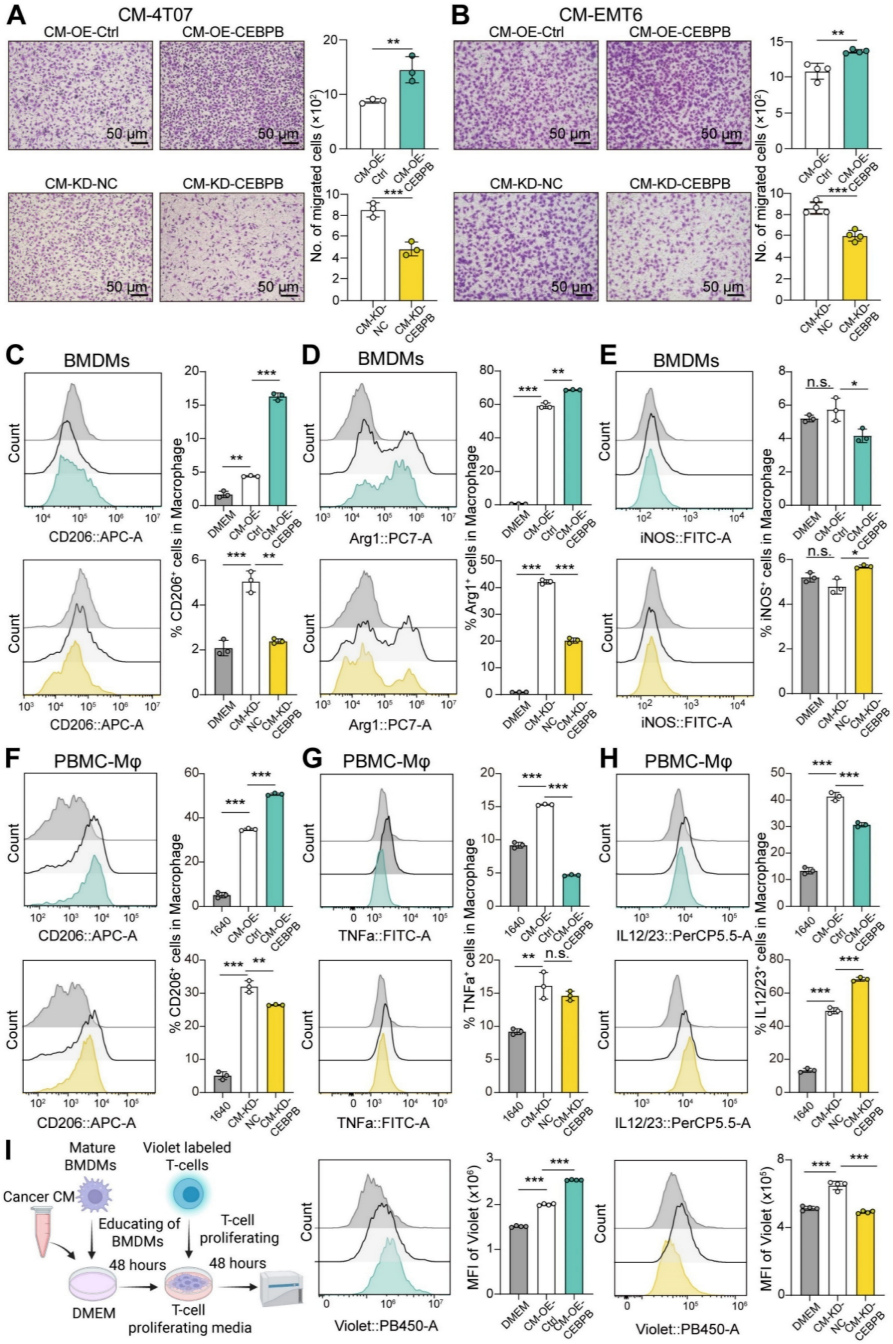
** Overexpression of CEBPB in TNBC cells promotes M2 Macrophage polarization, facilitating immune evasion. A-B**, Migration assay of BMDMs in conditional medium (CM) from 4T07 (A) or EMT6 (B) with CEBPB overexpression or knockdown. **C-E**, Percentages of CD206 (C), Arg1 (D), and iNOS (E) expressing BMDMs co-cultured with CM from 4T07 with CEBPB overexpression or knockdown. **F-H**, Percentages of CD206 (F), TNFa (G), and IL12/23 (H) expressing PBMC-Mφ co-cultured with CM from MDA-MB-231 with CEBPB overexpression or knockdown. **I**, Flow cytometry analysis for MFI of Violet dye in CD8^+^ T cells co-cultured with tumor cell derived CM-educated BMDMs, including CM from 4T07 with CEBPB overexpression or knockdown. n.s., not significant, *, *P* < 0.05, **, *P* < 0.01, ***, *P* < 0.001.

To investigate whether CEBPB expressed by tumor cells can recruit macrophages, we performed macrophage migration assays. Tumor cell derived conditional medium (CM) was collected from CEBPB overexpression tumor cells, as well as CEBPB knockdown tumor cells. CM was placed in the lower chamber of Transwell, while bone marrow-derived macrophages (BMDMs) were seeded into the upper chamber. After 24 hours of co-culture, we observed that incubation of CM-OE-CEBPB displayed a significantly stronger ability to recruit BMDMs compared to CM from control cells (**Figure 3A-B**). Conversely, incubation of CM-KD-CEBPB showed a reduced macrophage recruitment potential than the control group (**Figure 3A-B**).

Next, we explore whether CEBPB expression in tumor cells influences macrophage polarization. Macrophages were co-cultured with tumor cell derived CM for 48 hours. Flow cytometry analysis showed that incubation of CM-OE-CEBPB from 4T07 significantly increased the percentages of BMDMs expressing M2 markers CD206 and Arg1, while significantly reducing the proportion of BMDMs expressing M1 marker MHCII and iNOS, and inflammatory cytokines TNFa and IL12/23 compared to the control group. In contrast, incubation of CM-KD-CEBPB from 4T07 decreased the percentages of CD206 and Arg1 expressing, while slightly increasing the proportion of MHCII, iNOS, TNFa and IL12/23 expressing BMDMs (**Figure 3C-E and Figure S3A**). Similar experiments were conducted using human TNBC cell line MDA-MB-231 and human PBMC derived macrophages (PBMC-Mφ). Conditioned medium from MDA-MB-231 cells were collected and co-cultured with PBMC-Mφ, yielding similar results (**Figure 3F-H**). These results suggested that overexpression of CEBPB in TNBC tumor cells could recruit macrophage and induce M2 polarization *in vitro*.

Macrophages are widely recognized as a key immunosuppressive population within tumors, which can attenuate T cell responses 35. We then assessed the effect of the tumor CM-educated BMDMs on CD8^+^ T cell proliferation through cell co-culture. Prior to co-culture, CD8^+^ T cells were labeled with CellTrace Violet, a fluorescent dye that becomes diluted with each cell division, resulting in decreased fluorescence intensity. Flow cytometry analysis revealed that T cells co-cultured with tumor CM-educated BMDMs retained higher Violet fluorescence intensity compared to those co-cultured with DMEM-educated BMDMs, suggesting reduced T cell proliferation. Furthermore, BMDMs educated with CM-OE-CEBPB from 4T07 caused a higher Violet fluorescence intensity in T cells than the control group, indicating a significant decrease in T cell proliferation, whereas the opposite was observed with CM-KD-CEBPB from 4T07 (**Figure 3I**). These findings suggested that TNBC tumor cells overexpressing CEBPB can polarize macrophages towards to M2 phenotype, thereby inhibiting CD8^+^ T cell proliferation.

### Overexpression of CEBPB in tumors drive an immunosuppressive TME in TNBC

**Figure 4:**
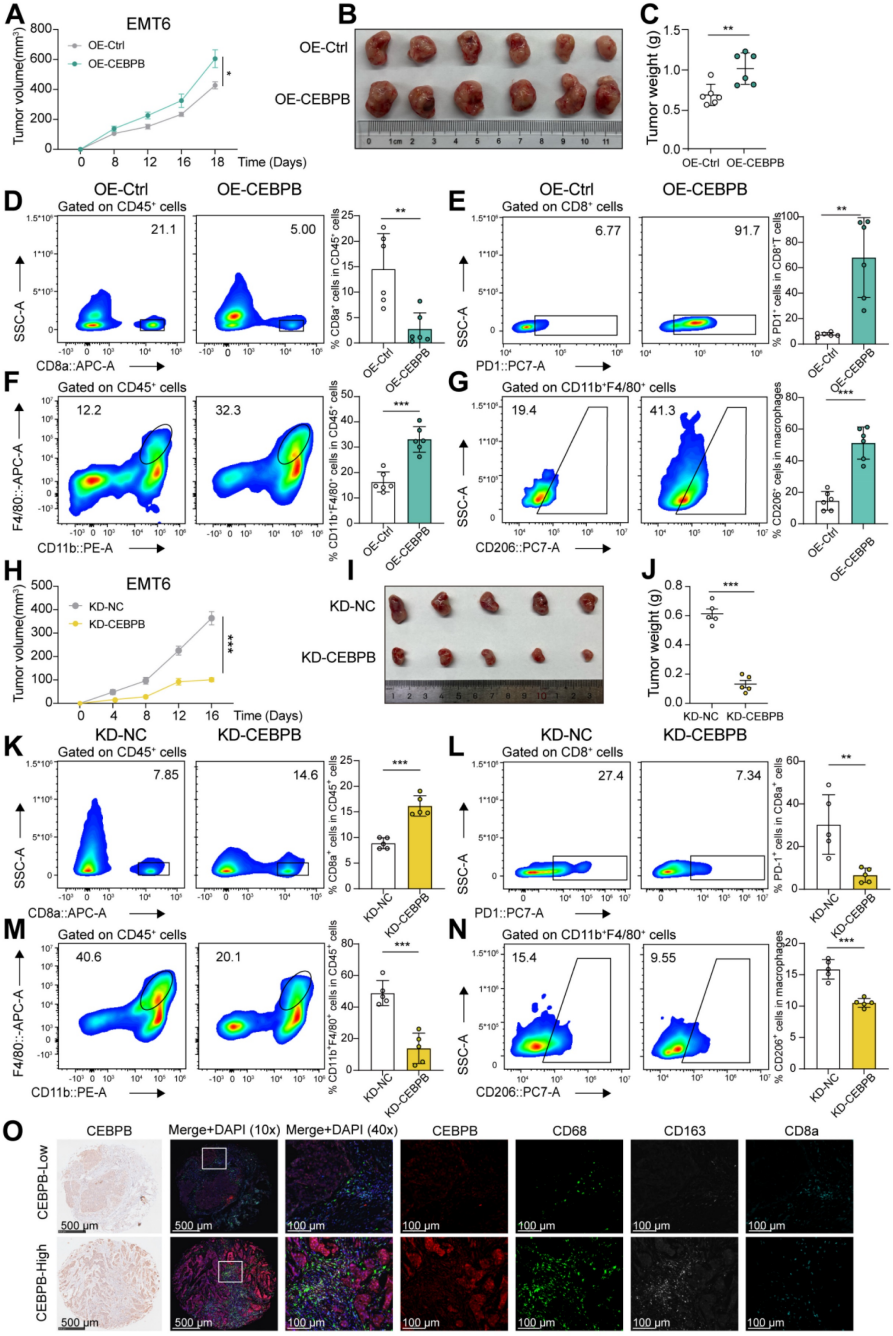
** Highly expressed CEBPB tumors facilitate a tumor-promoting phenotype in TNBC. A-C**, Tumor volume (A), image (B) and weight (C) of CEBPB-overexpressing (OE-CEBPB) or control (OE-Control) EMT6 allograts in BALB/c mice (*n* = 6). **D-G**, Flow cytometry analysis of CD8^+^ T cells (D), PD1^+^ CD8^+^ T cells (E), CD11b^+^ F4/80^+^ cells (F), and CD206^+^ macrophages (G) infiltrated in tumors developed in (A-C). **H-J**, Tumor volume (H), image (I) and weight (J) of CEBPB-knockdown (KD-CEBPB) or control (KD-NC) EMT6 allograts in BALB/c mice (*n* = 5). **K-N**, Flow cytometry analysis of CD8^+^ T cells (K), PD1^+^ CD8^+^ T cells (L), CD11b^+^ F4/80^+^ cells (M), and CD206^+^ macrophages (N) infiltrated in tumors developed in (H-J). **O**, Representative multiplex immunohistochemistry images of CEBPB, CD68, CD163, and CD8a staining in a tissue microarray (TMA) of TNBC patient tumor samples (*n* = 80). *, *P* < 0.05, **, *P* < 0.01, ***, *P* < 0.001.

We next evaluated the immunosuppressive role of CEBPB *in vivo*. CEBPB-overexpressing or control EMT6 cells were orthotopically implanted into the 4th pair of mammary fat pads in female BALB/c mice. We observed that overexpression of CEBPB promoted tumor growth in immunocompetent mice (**Figure 4A-C**). Considering that high CEBPB expression inhibited tumor cell proliferation *in vitro* and in allografts derived from nude mice, and our *in vitro* cell co-culture system demonstrated that elevated CEBPB expression tumor cells promote M2 macrophage polarization and suppress T cell proliferation, we hypothesized that the tumor-promoting effect of CEBPB might be immune-dependent. Therefore, we performed flow cytometry and immunohistochemistry staining analyses to assess changes in immune cell proportions within the tumors. Tumors overexpressing CEBPB had fewer CD8^+^ T cells, and a higher proportion of exhausted (PD-1^+^) CD8^+^ T cells compared with control tumors (**Figure 4D-E**). Additionally, high CEBPB expression tumors exhibited increased macrophages infiltration, particularly M2 macrophages (CD45^+^ CD11b^+^ F4/80^+^ CD206^+^) (**Figure 4F-G and Figure S3B-C**).

To confirm these findings, we also developed allografts derived from CEBPB-knockdown or control EMT6 cells. Tumor growth was significantly reduced in CEBPB-knockdown tumors compared to control tumors (**Figure 4H-J**). CEBPB knockdown tumors contained a significantly higher proportion of CD8^+^ T cells and a lower proportion of exhausted CD8^+^ T cells (**Figure 4K-L**). Moreover, the overall proportion of macrophages, particularly M2 macrophages, was markedly reduced in CEBPB knockdown tumors, consistent with observations from the high CEBPB expression tumors (**Figure 4M-N and Figure S3D**).

We then validated these results using multiplex immunohistochemistry on a TMA from 80 TNBC patients. We observed that CEBPB levels were positively correlated with the human macrophage marker CD68 and the M2 macrophage marker CD163, while being negatively correlated with the T cell marker CD8a (**Figure 4O and Figure S3E**).

We further performed IHC analyses in the corresponding allografts. In allografts with CEBPB overexpression, the expression level of the proliferation marker Ki67 was significantly lower compared with controls (**Figure S3C**). And there was no significant difference of Ki67 expression levels between CEBPB knockdown and the control tumors, possibly due to the smaller tumor size of knockdown tumors (**Figure S3D**). These results indicated a negative correlation between CEBPB and Ki67 expression in an immunocompetent tumor microenvironment. Therefore, we proposed that the pro-tumorigenic effect mediated by CEBPB is not attributable to a direct enhancement of tumor cell proliferation, but is more likely achieved through the induction of tumor cell dormancy, which may enhance immune evasion and ultimately manifest as an overall pro-tumor effect.

### The conditional knockdown of CEBPB enhances anti-tumor immunity and strengthens the therapeutic response to ICB

**Figure 5:**
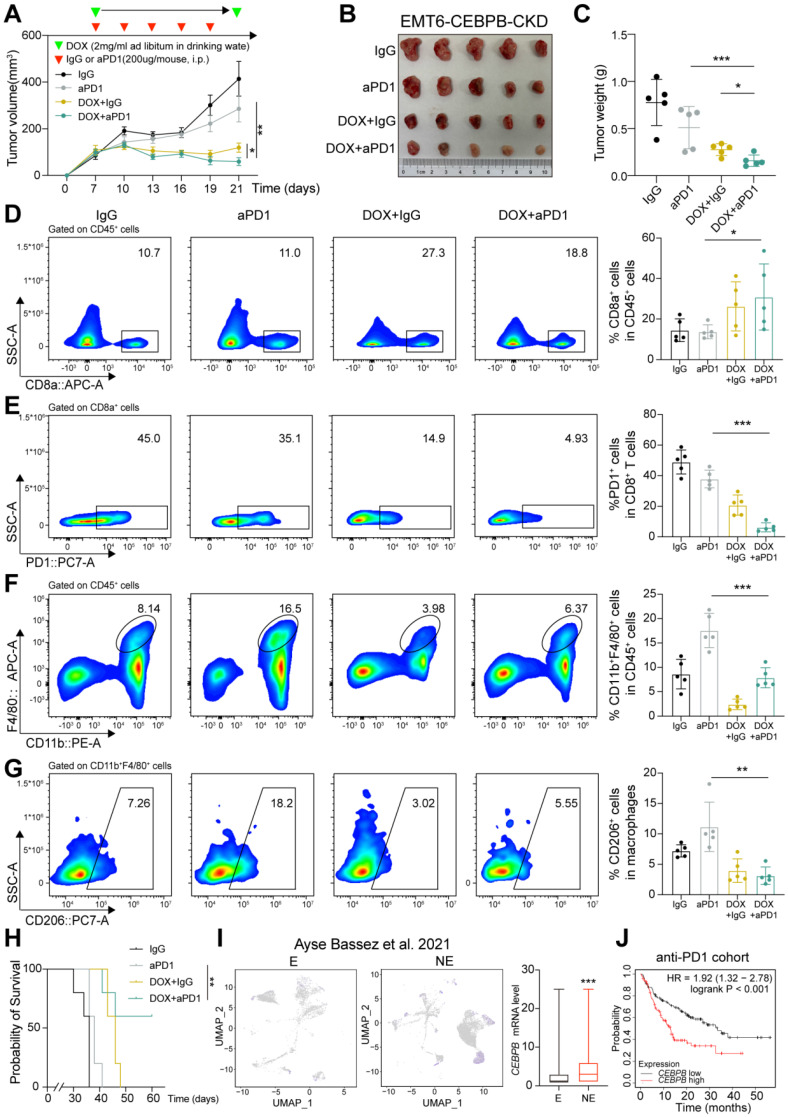
** The conditional knockdown of CEBPB boosts anti-tumor immunity and amplifies the therapeutic response to ICB. A-C**, Tumor volume (A), image (B) and weight (C) of EMT6 CEBPB-CKD allografts in BALB/c mice (*n* = 5). Tumor-bearing mice were randomly divided into four groups until tumor volume reached approximately 100 mm^3^. **D-G**, Flow cytometry analysis of CD8^+^ T cells (D), PD1^+^ CD8^+^ T cells (E), CD11b^+^ F4/80^+^ cells (F), and CD206^+^ macrophages (G) infiltrated in tumors developed of the indicated groups. **H**, Kaplan-Meier analysis of mice in each treatment group. **I**, Expression score of *CEBPB* in E and NE groups of tumor cells from 12 patients in the scRNA-seq data published by Ayse Bassez *et al.* 24. **J**, Kaplan-Meier analysis of *CEBPB* expression in a public cohort with patients receiving anti-PD-1 therapy. *, *P* < 0.05, **, *P* < 0.01, ***, *P* < 0.001.

To explore CEBPB expressed in tumor cells as a potential immunotherapy target for TNBC, we developed a tetracycline-responsive element-regulating conditional knockdown (CKD) lentiviral plasmid for CEBPB, simulating siRNA-mediated knockdown. We generated allografts derived from EMT6 -CKD cells, and divided them into four groups: IgG, anti-PD-1, DOX+IgG, and DOX+anti-PD-1 (**Figure 5A-C and Figure S3F**). The anti-PD-1 alone exhibited limited efficacy in suppressing tumor growth. Tumors treated with DOX alone resulted in tumor growth inhibition, and this effect was further enhanced in the combination treatment group (**Figure 5A-C**). Combination of anti-PD-1 with DOX therapy led to more CD8^+^ T cell infiltration and lower proportion of CD8^+^PD1^+^ T cells compared to the anti-PD-1 group (**Figure 5D-E**). Additionally, there was a reduction in macrophage infiltration, specifically M2 macrophages in the combination group (**Figure 5F-G**). Furthermore, mice received combination treatment experienced a significantly longer survival time compared to the anti-PD-1 therapy (**Figure 5H**).

We further found that *CEBPB* expression was significantly elevated in the anti-PD-1- NE group compared to the E group in the scRNA-seq data of tumor cells in 12 TNBC patients in Figure 1A (**Figure 5I**). Additionally, higher *CEBPB* expression was associated with poorer prognosis in a public dataset from 238 patients receiving anti-PD-1 therapy (**Figure 5J**). Therefore, our results suggest that targeting CEBPB may improve the efficacy of immunotherapy, potentially leading to better outcomes for TNBC patients.

### CEBPB induces M2 macrophage polarization through transcriptional activation of S100A8

**Figure 6:**
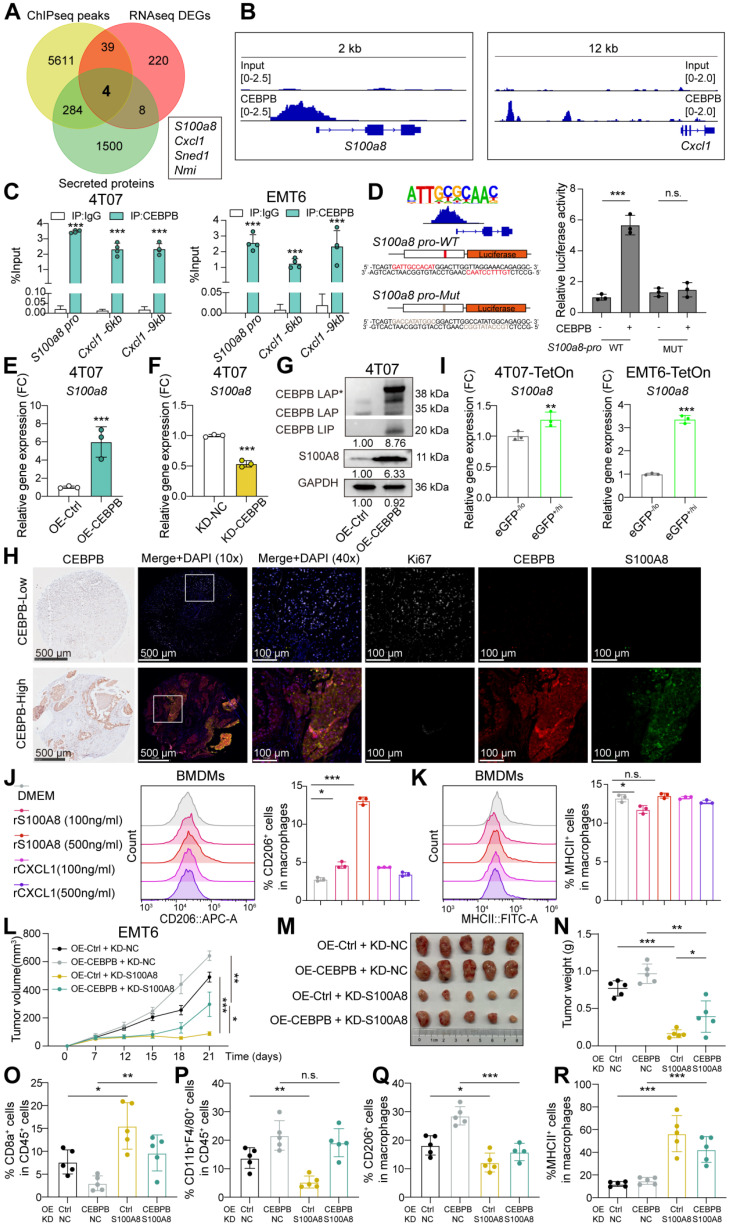
** CEBPB transcriptionally activates S100A8 to promote M2 macrophage polarization. A**, Venn diagram of three datasets identifying four common secretory factors: differentially expressed genes from CEBPB-overexpressing 4T07 cells bulk RNA-seq, CEBPB binding peaks in 4T07 cells from anti-CEBPB ChIP-seq, and the profile of mouse secretory proteins. **B**, CEBPB binding peaks for *S100A8* and *Cxcl1* from anti-CEBPB ChIP-seq in 4T07 cells. **C**, ChIP-qPCR showing the CEBPB binding regions in the *S100a8* promoter or upstream regions of *Cxcl1* of 4T07 and EMT6 cells. **D**, Relative luciferase activity in CEBPB-overexpressing HEK293T cells with the *S100a8* promoter WT or MUT, respectively. **E-F**, mRNA expression of *S100a8* or *Cxcl1* in 4T07 cells with CEBPB overexpression (E) or knockdown (F). **G**, Protein expression of S100A8 in 4T07 cells with or without CEBPB overexpression. **H**, Representative multiplex immunohistochemistry images of Ki67, CEBPB, and S100A8 staining in a TMA of TNBC patient tumor smaples (*n* = 80). **I**, mRNA expression of *S100a8* in eGFP^-/lo^ and eGFP^+/hi^ tumor cells sorted from 4T07-TetOn or EMT6-TetOn tumor masses. **J-K**, Percentages of CD206 (J) and MHCII (K) expressing BMDMs with the addition of rS100A8 (100 or 500 ng/mL) or rCXCL1 (100 or 500 ng/mL) for 48 hours. **L-N**, Tumor volume (L), image (M) and weight (N) of CEBPB-overexpressing with or without S100A8 knockdown EMT6 allografts in BALB/c mice (*n* = 5). **O-R**, Flow cytometry analysis of CD8^+^ T cells (O), CD11b^+^ F4/80^+^ cells (P), CD206^ +^ macrophages (Q), and MHCII^+^ macrophages (R) infiltrated in tumors developed in (L). n.s., not significant, *, *P* < 0.05, **, *P* < 0.01, ***, *P* < 0.001.

Recognizing the pivotal role of secretory molecules in macrophage polarization reprogramming, we aimed to identify potential secretory factors regulated by CEBPB that may mediate this effect. we firstly identified 271 differentially expressed genes (DEGs) based on the RNA-seq of OE-CEBPB vs OE-Ctrl 4T07 cells (**Table S4**). A total of 5,938 genes from potential CEBPB binding peaks were identified from the anti-CEBPB ChIP-seq in 4T07 (**Table S5**). Additionally, 1,796 mouse-derived secretory factors were derived from CellChat, which offers the latest and most comprehensive collection of ligand-receptor pair information for cell-cell interaction analysis in scRNA-seq 40. By intersecting the DEGs, ChIP peaks, and mouse secretory factors, we identified four common genes: *S100a8*, *Cxcl1*, *Nmi*, and *Sned1* (**Figure 6A**). Notably, we observed robust CEBPB binding in the *S100a8* promoter region, and 6 kb or 9 kb upstream region of the *Cxcl1* transcription start site, indicating that S100A8 and CXCL1 are likely downstream targeting secretory factors regulated by CEBPB (**Figure 6B and Figure S4A**).

To verify these findings, we performed ChIP-qPCR and DNA gel electrophoresis. Significant CEBPB binding enrichments were confirmed in the promoter region of *S100a8*, and upstream regions of *Cxcl1* (**Figure 6C**). Additionally, CEBPB binding enrichment further increased after CEBPB overexpression (**Figure S4B-E**). These findings demonstrate that CEBPB specifically binds to these regions, particularly the *S100a8* promoter. Using JASPAR database, we predicted two major CEBPB binding sites within the CEBPB binding region in *S100a8* promoter, each located near the peak center on two different strands of DNA. Dual-luciferase reporter assays confirmed that these two binding sites are crucial for the transcriptional regulation by CEBPB. We cloned the DNA sequences of *S100a8* promoter into firefly luciferase reporter plasmid, and constructed a variant with CEBPB binding site mutation. CEBPB overexpression significantly enhanced the relative luciferase activity of *S100a8* promoter, while the binding site mutation made CEBPB lost its regulatory ability (**Figure 6D**). Subsequent qPCR analysis confirmed that overexpression of CEBPB significantly upregulated the mRNA levels of *S100a8* and *Cxcl1*, while CEBPB knockdown reduced their transcription (**Figure 6E-F and Figure S4F-G**). Western blot analysis further demonstrated that CEBPB promoted S100A8 protein level (**Figure 6G and Figure S4H**). The multiplex immunohistochemistry experiment further revealed a strong positive correlation between CEBPB and S100A8 expression in 80 TNBC tumor tissues from a TMA. We also observed a negative correlation between CEBPB and Ki67 expression in the TMA, consistent with our finding that high CEBPB expression is associated with tumor dormancy (**Figure 6H and Figure S4I**). Finally, we found that S100A8 was significantly overexpressed in dormant tumor cells sorted from both 4T07-TetOn and EMT6-TetOn allografts (**Figure 6I**). Collectively, these results demonstrate that CEBPB specifically activates the transcription of S100A8 and CXCL1.

We then explored the functional roles of S100A8 and CXCL1 in macrophage polarization. Incubation with recombinant mouse S100A8 (rS100A8) or CXCL1 (rCXCL1) protein markedly increased the proportion of CD206^+^ BMDMs, with rS100A8 showing a stronger effect with a concentration-dependent manner (**Figure 6J and Figure S5A**). Meanwhile, rS100A8 reduced the percentages of MHCII^+^ or iNOS^+^ BMDMs, whereas rCXCL1 showed no significant effect (**Figure 6K and Figure S5B**). In total, these results indicate that S100A8 is a more potent inducer of M2 macrophage polarization.

Next, we found that S100A8 knockdown reduced macrophage recruitment and promoted their M1 polarization in EMT6 allografts, in line with our *in vitro* results (**Figure 6L-R and Figure 5C-E**). Meanwhile, the infiltration of CD8^+^ T cells was increased, and the proportion of exhausted T cells was decreased. More importantly, S100A8 knockdown reversed the restricted CD8^+^ T infiltration and increased exhaustion of CD8^+^ T cells induced by CEBPB overexpression (**Figure 6O and Figure S5E**). Although S100A8 knockdown did not reduced macrophage infiltration caused by CEBPB overexpression, it decreased the proportion of M2 macrophage and increased M1 macrophage fraction (**Figure 6P-R and Figure S5E**). Our data demonstrate that S100A8 can promote M2 macrophage polarization. Also, targeting S100A8 may improve the immune-suppressive TME caused by CEBPB overexpression.

### Inhibition of S100A8 remodels TME and enhances ICB efficacy

**Figure 7:**
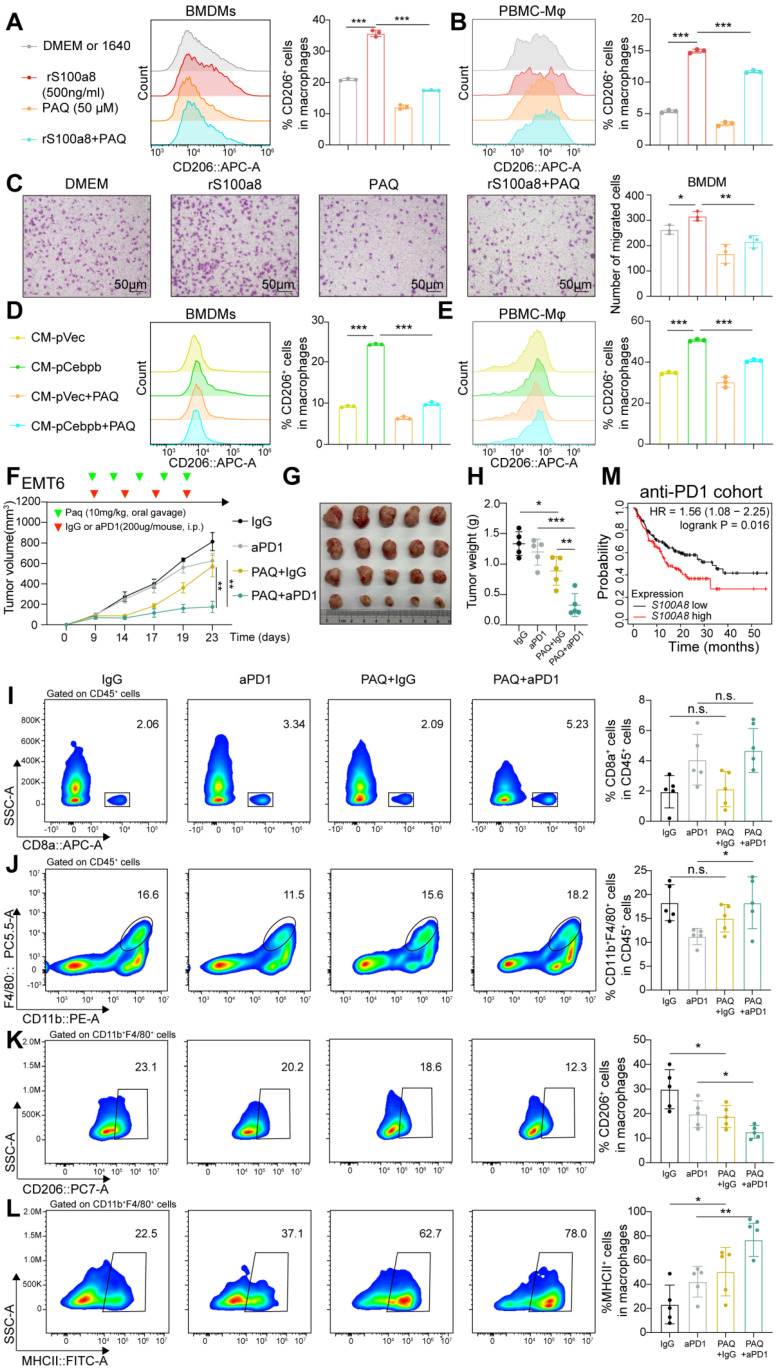
** The S100A8 inhibitor remodels TME and improves ICB efficacy. A-B**, Percentages of CD206 expressing in BMDMs (A) or PBMC-Mφ (B) with the addition of paquinimod (PAQ, 5 µg/mL) and/or rS100A8 (500 ng/mL) for 48 hours. **C**, Migration assay of BMDMs in DMEM with the addition of PAQ (5 µg/mL) and/or rS100A8 (500 ng/mL) for 24 hours. **D-E**, Percentages of CD206 expressing in BMDMs (D) or PBMC-Mφ (E) co-cultured with CM and/or PAQ (5 µg/mL) for 48 hours, including CM from 4T07 (D) or MDA-MB-231 (E) with or without CEBPB overexpression. **F-H**, Tumor volume (F), image (G) and weight (H) of EMT6 allografts in BALB/c mice (*n* = 5). Tumor-bearing mice were randomly divided into four groups until tumor volume reached approximately 100 mm^3^. **I-L**, Flow cytometry analysis of CD8^+^ T cells (I), CD11b^+^ F4/80^+^ macrophages (J), CD206^+^ macrophages (K) MHCII^+^ macrophages (L) infiltrated in tumors developed of the indicated groups. **M**, Kaplan-Meier analysis of *S100A8* expression in a public cohort with 238 patients receiving anti-PD-1 therapy. n.s., not significant, *, *P* < 0.05, **, *P* < 0.01, ***, *P* < 0.001**.**

Since extracellular S100A8 exerts its biological functions by binding to specific receptors, we next explored whether pharmacological inhibitors blocking the S100A8 -receptor interaction could impact macrophage reprogramming, thus modulating the TME. Paquinimod (PAQ) functions by directly blocking the hydrophobic cleft structure of S100A8 involved in receptor recognition 41. We co-treated BMDMs with rS100A8 and/or PAQ for 48 hours. Compared to rS100A8 alone, the combined application of PAQ and rS100A8 elicited a marked reduction in the percentages of CD206^+^ BMDMs or CD206^+^ PBMC-Mφ cells, while paradoxically enhancing the proportions of MHCII^+^, iNOS^+^, or TNFa^+^ BMDMs (**Figure 7A-B and Figure S6A-C**). It also markedly reduced the recruitment of BMDMs induced by rS100A8 (**Figure 7C**). In addition, we co-treated BMDMs with PAQ and CM from CM-OE-CEBPB or control tumor cells for 48 hours. We found that PAQ rescued the proportions of CD206, MHCII, and TNFa expression in either BMDMs or PBMC-Mφ, induced by CM from CEBPB overexpression tumor cells (**Figure 7D-E and Figure S6D-G**). These above results indicate that S100A8 inhibitor PAQ may reduce macrophage recruitment and M2 macrophage polarization.

We further evaluated the efficacy of combining PAQ with anti-PD-1 therapy. The combination of these two agents resulted in significantly reduced tumor mass compared to either treatment alone (**Figure 7F-H**). Compared to the anti-PD-1 monotherapy group, the PAQ+IgG group did not show increased CD8^+^ T cell infiltration, while the combination therapy group exhibited more CD8^+^ T cell infiltration, reduced the proportion of M2 macrophages, and increased the proportion of M1 macrophages (**Figure 7I-L**). In addition, higher *S100A8* expression was also associated with poorer prognosis in a public anti-PD-1 therapy dataset of 238 patients (**Figure 7M**). These findings suggest that PAQ may serve as a promising way to enhance immunotherapy for TNBC, potentially by reprogramming M2 macrophages into an M1 phenotype.

In addition, we performed IHC staining for Ki67 and CEBPB. Compared with control tumors, anti-PD-1 therapy markedly reduced Ki67 expression and increased CEBPB levels, thereby recapitulating a niche enriched for dormant tumor cells. Combined treatment with anti-PD-1 and PAQ led to a pronounced reduction in both Ki67 and CEBPB expression (**Figure S6H**). Collectively, these results support the notion that tumors enriched for dormant tumor cells may be better candidates for S100A8 inhibitor therapy.

## Discussion

Dormant tumor cells have been identified as immunotherapy-resistant reservoirs**,** potentially interacting with tumor-associated macrophages (TAMs) within their immune-privileged niches 5,6. Investigation of macrophages infiltration and phenotype within these niches may reveal the mechanisms underlying immune checkpoint blockade (ICB) resistance. We found that dormant tumor cells with high CEBPB expression promote macrophage infiltration and M2 polarization through the secretion of S100A8, leading to immune evasion. Therefore, blocking S100A8 from binding to its receptor may serve as a promising therapeutic strategy to potentiate ICB efficacy in TNBC.

We investigated the role of CEBPB in tumor dormancy, as CEBPB is the commonly upregulated genes in both long-term dormant tumor cells (label-retention method) and transient dormant tumor cells (mVenus-p27K^-^ reporter). CEBPB is a member of the C/EBP transcription factor family, and has been reported to negatively regulate the cell cycle in cancers 36,37. In Ras-induced senescence of mouse embryo fibroblasts, CEBPB interacts with the Rb-E2F complex to suppress the expression of G1/S phase-related genes 38,39. Here, we also found that overexpression of CEBPB induced G1-phase arrest in TNBC cells. We identified prominent CEBPB binding peaks at the promoter regions of cell cycle-related gene *Ccng2*, and enhancer region of *Cdkn1b*. It has been reported that CCNG2 blocks the G1/S phase transition by inhibiting the phosphorylation of CDK2 and Rb 42,43. p27 (encoded by *Cdkn1b*) is a well-known cyclin-dependent kinase inhibitor, which inhibits cell cycle progression through both the G0/G1 and G1/S transitions 44,45. Thus, we found that high CEBPB expression in dormant tumor cells promotes CCNG2 and p27 expression to maintain tumor dormancy.

In addition to tumor dormancy maintenance, CEBPB plays a crucial role in driving an immunosuppressive tumor phenotype, characterized by increased M2 macrophage infiltration and a reduced CD8^+^ T cell population in TNBC. Macrophage polarization within tumors is primarily regulated by properties of tumor microenvironment (TME) like local hypoxia, fibrosis, cell stress, and secreted molecules.

Based on our co-culture experiments using tumor cell-conditioned medium and macrophages *in vitro*, we proposed that CEBPB modulates the secretory profile of dormant tumor cells, thereby promoting M2 macrophage polarization. Furthermore, we found that CEBPB directly regulates the secretory protein S100A8 at multiple levels, including transcription factor binding, transcription, and protein levels, consistent with previous reports 46. Extracellular S100A8 is an immunosuppressive cytokine known to promote the accumulation of myeloid-derived suppressor cells or TAMs in the pre-metastatic niche of breast cancer 47-49. Besides promoting macrophages migration, S100A8 also facilitates M2 macrophage polarization in cancer 50. Notably, S100A8 functions as a downstream target of CEBPB in immune evasion. S100A8 knockdown significantly reduced M2 macrophage infiltration induced by CEBPB overexpression in allografts. Thus, we demonstrated that CEBPB-high dormant tumor cells promote immune evasion through S100A8 secretion, thereby preserving the immune-privileged niche.

The ICB efficacy is determined by the TME. Drugs that prevent immunosuppression or enhance anti-tumoral immune activity may improve ICB response. The doxycycline-induced CEBPB knockdown model, simulating siRNA-based CEBPB targeting, exhibited enhanced anti-PD-1 efficacy in terms of reduced tumor growth, longer survival, and TME remodeling. It should be noted that the efficacy of siRNA targeting CEBPB depend on factors such as drug safety and tumor-specific delivery. Therefore, we focused on the CEBPB downstream target S100A8, which can be inhibited by some known compounds. Among them, paquinimod (PAQ), a quinoline-3-carboxamide derivative, has been found to directly block S100A8 binding to its receptors TLR4 or RAGE 41. While PAQ has been participated in clinical trials for various autoimmune diseases, research on its application in cancer treatment remains limited 51-53. We observed that while PAQ alone has minimal effects on tumor growth, it markedly enhances anti-PD-1 efficacy by reprogramming M2 macrophages to the M1 phenotype and increasing CD8^+^ T cell infiltration in TNBC allografts. Therefore, we consider PAQ as an effective agent for remodeling TME.

Notably, we found that high expression of CEBPB and S100A8 is associated with poor response to PD-1 therapy in cancer patients, and mIHC analysis of TMA samples further confirmed a positive correlation between CEBPB and S100A8 expression in TNBC patients. These findings link ICB resistance with elevated CEBPB and S100A8 expression in TNBC, and suggest that TNBC patients enriched with dormant tumor cells, particularly those characterized by high CEBPB expression, are more likely to benefit from PAQ treatment. Furthermore, compared with anti-PD-1 monotherapy, combined PAQ and anti-PD-1 treatment resulted in reduced Ki67 and CEBPB expression, possibly due to disruption of a dormancy-dominated TME by PAQ, leading to enhanced elimination of tumor cells, including dormant tumor cells, and consequent tumor regression. Given its oral administration and minimal side effects, S100A8 inhibitor-PAQ warrants further clinical exploration in combination with ICB in TNBC patients.

This study has several limitations that should be acknowledged. As a key factor of dormant tumor cells in TNBC, the mechanisms driving CEBPB upregulation in this context remain to be elucidated. And the CEBPB-overexpression model used to represent dormant tumor cells is simplified and may not fully capture CEBPB's role in the complex regulatory network of tumor dormancy. Also, our investigation of the relationship between dormant tumor cells and the TME primarily focused on the secretory phenotype, leaving unexplored factors such as hypoxia and angiogenesis, which are crucial aspects of the dormant state. Moreover, while this study identified S100A8 as a factor in M2 macrophage polarization, the exact molecular mechanisms of this polarization reprogramming remain unclear. In addition, whether the conclusions drawn from TNBC can be generalized to other cancer types requires additional supporting evidence. These limitations suggest avenues for future research to gain a more comprehensive understanding of tumor dormancy.

## Conclusions

In summary, this study establishes CEBPB as a central regulator connecting tumor dormancy and immune evasion in TNBC. We demonstrate that dormant tumor cells, characterized by elevated CEBPB expression, constitute an immunotherapy-resistant reservoir. Mechanistically, CEBPB enforces a dormant phenotype by directly transactivating key cell cycle inhibitor CCNG2, inducing G1-phase arrest. Concurrently, it orchestrates an immunosuppressive TME by regulating the secretory phenotype of dormant tumor cells, particularly through S100A8, which in turn recruits and polarizes tumor-associated macrophages toward an M2 phenotype, thereby creating an immune-privileged niche conducive to tumor cell survival and resistance to anti-PD-1 therapy. Importantly, targeting this axis with the S100A8 inhibitor paquinimod effectively remodels the TME, reverting M2 macrophage polarization and enhancing CD8^+^ T cell infiltration, which synergizes with anti-PD-1 to suppress tumor growth. These findings not only elucidate a direct molecular link between dormancy maintenance and immune evasion but also propose a novel and translatable therapeutic strategy to target the dormant cell reservoir and overcome ICB resistance in TNBC.

## Supplementary Material

Supplementary figures.

Supplementary tables.

## Data Availability

Publicly available data analyzed in this study—the RNA-seq data were obtained from GEO at GSE198713 and GSE267757. Read counts of scRNA-seq data from Ayse Bassez *et al.* [24] were accessed publicly via biokey.lambrechtslab.org. the profile of secretory factors was obtained from CellChat (http://www.cellchat.org/cellchatdb/). All data, code, and materials are available upon request. Raw data of CEBPB-overexpressing RNA-seq and anti-CEBPB ChIP-seq are available from GEO under accession number GSE290038 and GSE290039. In addition, the survival analysis of CEBPB and S100A8 in the public anti-PD-1 dataset of 238 patients derived from the Kaplan-Meier Plotter platform [27].

## Abbreviations

ICB: Immune checkpoint blockade
TNBC: Triple-negative breast cancer
TAMs: Tumor-associated macrophages
CEBPB: CCAAT/enhancer-binding protein beta
TME: Tumor microenvironment
DOX: Doxycycline
TMA: Tissue microarray
CM: Conditional medium
CKD: Conditional knockdown
BMDMs: Bone marrow-derived macrophages
PBMC-Mφ: Peripheral blood mononuclear cell-derived macrophages
PAQ: Paquinimod
