# Supplementary material for: CEBPB-high dormant tumor cells drive immune evasion via S100A8 orchestrated tumor-associated macrophages reprogramming
Source: Theranostics. 2026 Feb 26;16(9):4883–904. doi: 10.7150/thno.124789 (PMC12964385; doi:10.7150/thno.124789)
Supplement: Supplementary file 1 — Supplementary figures. [file thnov16p4883s1.pdf]

CEBPB-high dormant tumor cells drive immune evasion via S100A8 orchestrated tumor-associated macrophages reprogramming

Jin Bai [1, 2], Huilan Su [3], Shunxi Wang[4,5*], Yang Dong [3,4*], Yong Gao [1*]

Affiliation

1 Department of Oncology, Shanghai East Hospital, Tongji University School of Medicine, 150 Jimo Road, Pudong District, Shanghai, 200120, China.

2 Cancer Center of Daping Hospital, Army Medical University, Chongqing, 400037, China.

3 Research Center for Translational Medicine, Cancer Stem Cell Institute, Shanghai East Hospital, Tongji University School of Medicine, 150 Jimo Road, Pudong District, Shanghai, 200120, China.

4 Institute of Pathology and Southwest Cancer Center, Southwest Hospital, Third Military Medical University (Army Medical University), and The Key Laboratory of Tumor Immunopathology, The Ministry of Education of China, Chongqing, 400038, P.R. China

5 Chongqing Institute of Advanced Pathology, Jinfeng Laboratory, Chongqing, 400039.China

*Corresponding author

Yong Gao (drgaoyong@tongji.edu.cn)

Yang Dong (1810441@tongji.edu.cn)

Shunxi Wang (sx_wang1024@126.com)

Supplementary Figures

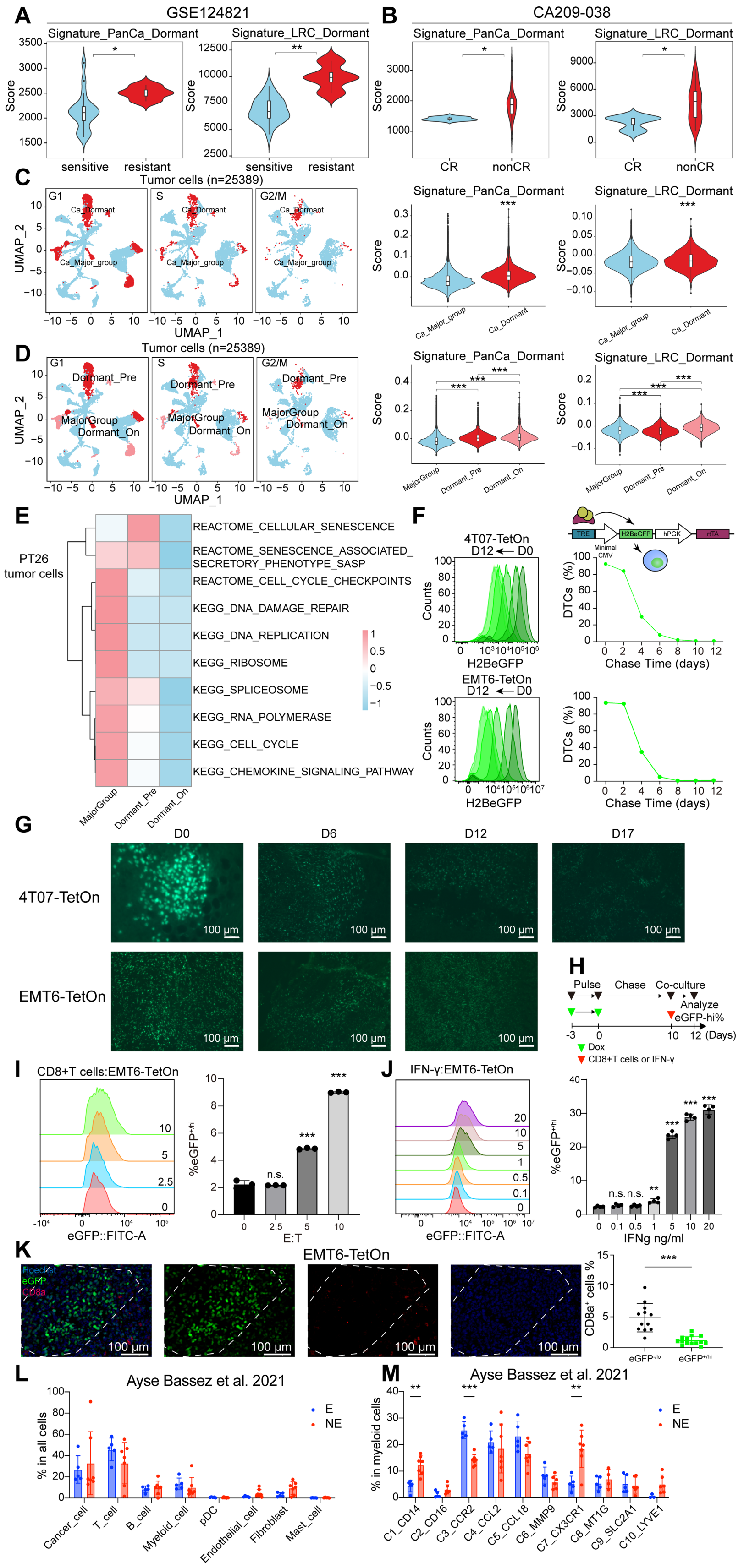

Figure S1. Dormant tumor cells occupy an immunosuppressive niche and mediate ICB resistance in TNBC.

A-B, Violin plots showing expression scores of Signature_LRC_Dormant and Signature_PanCa_Dormant in GSE124821 and RNA-seq data of the CA209-038 study.

C, Umap plots of cell cycle score analysis categorizing 25389 tumor cells from the all 12 TNBC patients into Dormant and MajorGroup clusters (Left). Violin plots showing expression scores of Signature_LRC_Dormant and Signature_PanCa_Dormant in these clusters (Right).

D, Umap plots of cell cycle score analysis categorizing 25389 tumor cells from the all 12 TNBC patients into Dormant_Pre, Dormant_On, and MajorGroup clusters (Left). Violin plots showing expression scores of Signature_LRC_Dormant and Signature_PanCa_Dormant in these clusters (Right).

E, GSVA analysis of tumor cell clusters from PT26 patient.

F, Label retention effect of TetOn-H2BeGFP system on 4T07 and EMT6 cells cultured in vitro. 4T07-TetOn and EMT6-TetOn cells were cultured in doxycycline (DOX) -containing medium (5 μg/mL) for 3 days (pulsing), followed by passaging in DOX-free medium for 12 days (chasing). Cells were collected every 2 days, and the proportion of cells retaining eGFP fluorescence was analyzed using flow cytometry (left). Fluorescence retention curves for the 12-day chase period were also generated (right), demonstrating that after 8-12 days of chasing, only approximately 1% of the cells retained elevated levels of eGFP fluorescence, thereby identifying them as dormant tumor cells.

G, The TetOn-H2BeGFP system demonstrated the labeling retention effect in the in vivo tumors. The retention of eGFP fluorescence in 4T07-TetOn and EMT6-TetOn allografts were assessed using fluorescence microscopy.

H, Experimental model diagram of T cell or IFN-γ mediated killing assay.

I, T cell cytotoxicity assay. $CD8^+$ T cells from OT-1 mice were co-cultured with EMT6-TetOn-OVA cells that had been labeled with DOX and tracked for 10 days at varying effector to target ratios (E:T) for 48 hours.

J, IFN-γ cytotoxicity assay, EMT6-TetOn cells labeled with doxycycline and tracked for 10 days were treated with different concentrations of IFN-γ for 48 hours. The proportion of $eGFP^{+/high}$ cells was analyzed using flow cytometry.

K, Representative IF plots of CD8a staining in EMT6-TetOn serial tumor sections. White dashed line marks $eGFP^{+/high}$ area.

L, The percentage of major cell subpopulation refers to its proportion out of the patient's total cells from a cohort of 12 TNBC patients in the scRNA-seq dataset published by Ayse Bassez et al., categorized into clonotype expansion (E, blue) and no/limited expansion (NE, red) groups based on T cell clone expansion following anti-PD-1 therapy.

M, The percentage of macrophage subpopulation refers to its proportion out of the patient's main macrophage count from a cohort of 12 TNBC patients in the scRNA-seq dataset published by Ayse Bassez et al., categorized into clonotype expansion (E, blue) and no/limited expansion (NE, red) groups based on T cell clone expansion following anti-PD-1 therapy. n.s, not significant, *, $P < 0.05$, **, $P < 0.01$, ***, $P < 0.001$.

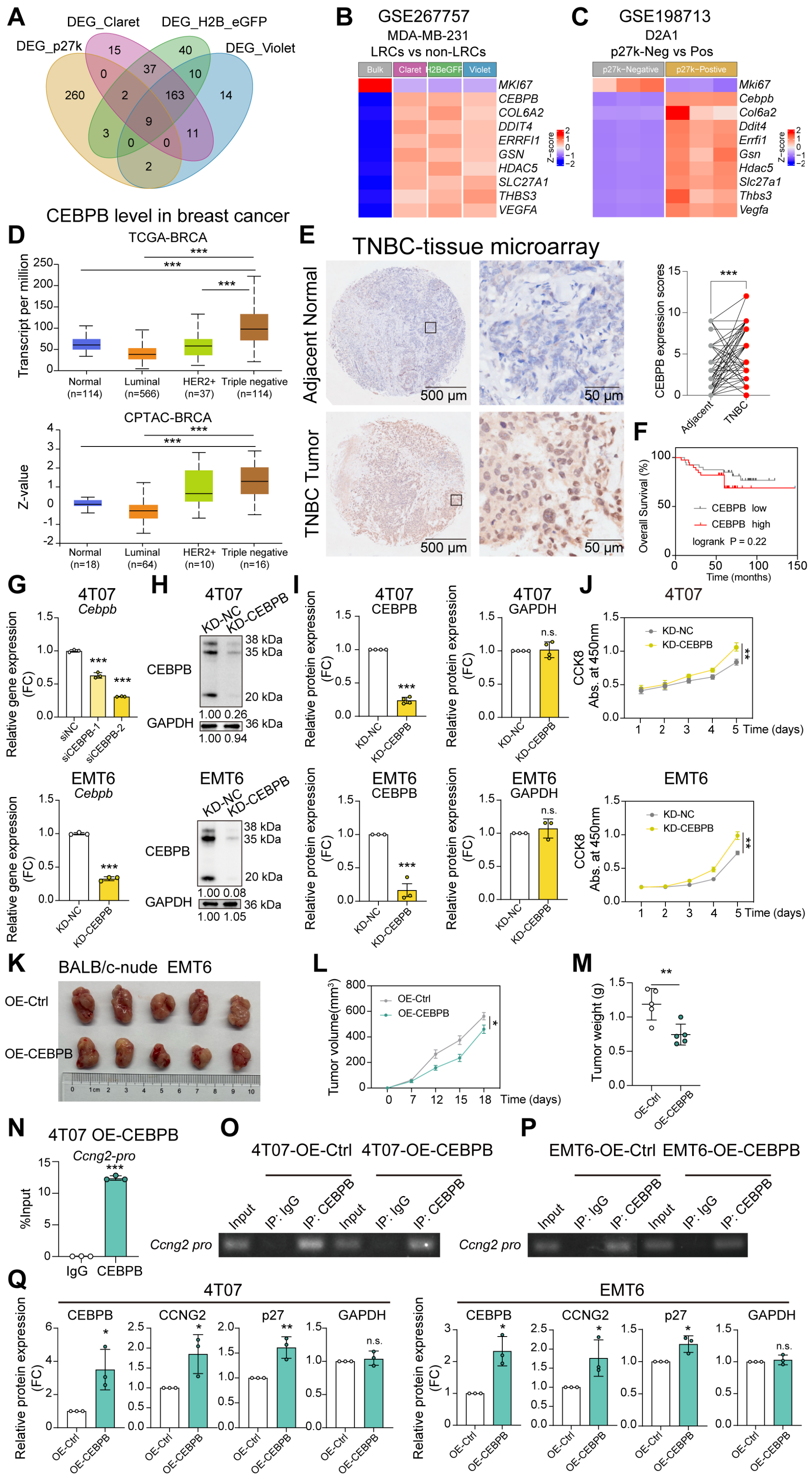
A
DEG_Claret
DEG_H2B_eGFP
DEG_p27k
DEG_Violet
15
40
0
37
10
260
2
163
14
9
3
11
0
0
2
B
GSE267757
MDA-MB-231
LRCs vs non-LRCs
Bulk
Claret
H2BeGFP
Violet
MKI67
CEBPB
COL6A2
DDIT4
ERRFI1
GSN
HDAC5
SLC27A1
THBS3
VEGFA
Z-score
C
GSE198713
D2A1
p27k-Neg vs Pos
p27k−Negative
p27k−Postive
Mki67
Cebpb
Col6a2
Ddit4
Errfi1
Gsn
Hdac5
Slc27a1
Thbs3
Vegfa
Z-score
CEBPB level in breast cancer
D
TCGA-BRCA
Transcript per million
Normal (n=114)
Luminal (n=566)
HER2+ (n=37)
Triple negative (n=114)
CPTAC-BRCA
Z-value
Normal (n=18)
Luminal (n=64)
HER2+ (n=10)
Triple negative (n=16)
E
TNBC-tissue microarray
Adjacent Normal
TNBC Tumor
500 μm
50 μm
CEBPB expression scores
Adjacent
TNBC
F
Overall Survival (%)
CEBPB low
CEBPB high
logrank P = 0.22
Time (months)
G
4T07
Cebpb
Relative gene expression (FC)
siNC
siCEBPB-1
siCEBPB-2
EMT6
Cebpb
KD-NC
KD-CEBPB
H
4T07
KD-NC
KD-CEBPB
CEBPB
GAPDH
38 kDa
35 kDa
20 kDa
36 kDa
1.00 0.26
1.00 0.94
EMT6
1.00 0.08
1.00 1.05
I
4T07
CEBPB
GAPDH
Relative protein expression (FC)
EMT6
n.s.
J
4T07
CCK8
Abs. at 450nm
KD-NC
KD-CEBPB
Time (days)
EMT6
K
BALB/c-nude EMT6
OE-Ctrl
OE-CEBPB
L
Tumor volume(mm³)
OE-Ctrl
OE-CEBPB
Time (days)
M
Tumor weight (g)
OE-Ctrl
OE-CEBPB
N
4T07 OE-CEBPB
Ccng2-pro
%Input
IgG
CEBPB
O
4T07-OE-Ctrl
4T07-OE-CEBPB
Input
IP: IgG
IP: CEBPB
Ccng2 pro
P
EMT6-OE-Ctrl
EMT6-OE-CEBPB
Input
IP: IgG
IP: CEBPB
Ccng2 pro
Q
4T07
EMT6
CEBPB
CCNG2
p27
GAPDH
Relative protein expression (FC)
OE-Ctrl
OE-CEBPB
n.s.

Figure S2. CEBPB as an essential factor in the tumor dormancy of TNBC Cells.

A, Venn diagram showing the upregulated genes in dormant TNBC cells identified from GSE267757 and GSE198713.

B-C, Heatmaps showing the 9 common upregulated genes and *Ki67* mRNA expression in GSE267757 (B) and GSE198713 (C).

D, boxplot of CEBPB expression levels across different breast cancer subtypes from TCGA-BRCA and CPTAC-BRCA.

E, Representative immunohistochemistry images of CEBPB staining in a TMA with adjacent normal samples and TNBC tumor samples (n = 80 per group), and the expression of CEBPB protein in the two groups was statistically analyzed.

F, Kaplan-Meier analysis (log-rank test) of CEBPB expression in tumor samples of the TNBC-TMA. Median expression of CEBPB was as the group cutoff (n = 40 per group).

G, mRNA expression of *Cebpb* in 4T07 cells transfected with the siCEBPB-1 and siCEBPB-2. The siCEBPB-2 sequence was selected to construct the KD-CEBPB lentivirus vector. And mRNA expression of *Cebpb* in EMT6 KD-CEBPB cells.

H-I, Protein expression of CEBPB in 4T07 KD-CEBPB and EMT6 KD-CEBPB cells.

J, Cell viability assessed by Cell Counting Kit-8 assay in 4T07 and EMT6 cells with or without CEBPB knockdown.

K-M, Tumor image (K), volume (L) and weight (M) of EMT6 OE-CEBPB allografts in BALB/nude mice (n = 5).

N-P, ChIP-qPCR (N) and DNA gel electrophoresis (O-P) showing the CEBPB binding region in the promoter regions of *Ccng2* of 4T07 and EMT6 cells with or without CEBPB overexpression.

Q, Corresponding statistic data of Figure2M. *, $P < 0.05$, **, $P < 0.01$, ***, $P < 0.001$.

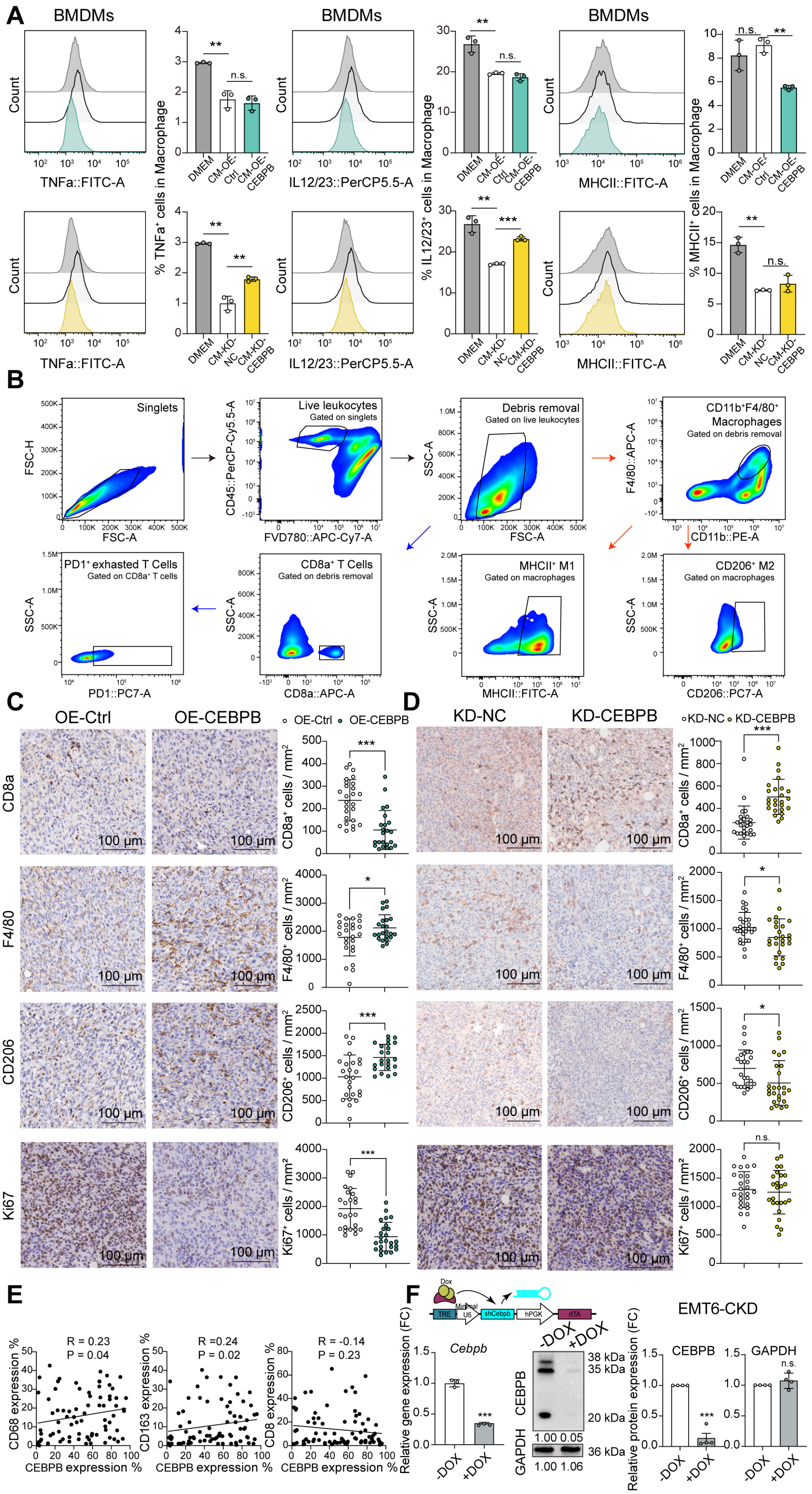
A
BMDMs
TNFa::FITC-A
IL12/23::PerCP5.5-A
MHCII::FITC-A
% TNFa⁺ cells in Macrophage
% IL12/23⁺ cells in Macrophage
% MHCII⁺ cells in Macrophage
DMEM
CM-OE-Ctrl
CM-OE-CEBPB
CM-KD-NC
CM-KD-CEBPB
B
Singlets
Live leukocytes
Gated on singlets
Debris removal
Gated on live leukocytes
CD11b⁺F4/80⁺ Macrophages
Gated on debris removal
PD1⁺ exhausted T Cells
Gated on CD8a⁺ T cells
CD8a⁺ T Cells
Gated on debris removal
MHCII⁺ M1
Gated on macrophages
CD206⁺ M2
Gated on macrophages
C
OE-Ctrl
OE-CEBPB
CD8a
F4/80
CD206
Ki67
D
KD-NC
KD-CEBPB
E
R = 0.23
P = 0.04
R =0.24
P = 0.02
R = -0.14
P = 0.23
CEBPB expression %
F
EMT6-CKD
Cebpb
CEBPB
GAPDH
-DOX
+DOX

Figure S3. Highly expressed CEBPB tumors facilitate a tumor-promoting phenotype in TNBC.

A, Percentages of TNFa, IL12/23 and MHC II expressing BMDM co-cultured with CM from 4T07 with CEBPB overexpression or knockdown.

B, Gating strategy for tumor infiltrating immune cells

C-D, Representative immunohistochemistry images of CD8a, F4/80, CD206 and Ki67 staining in serial tumor sections of EMT6 allografts from CEBPB overexpression (C) or knockdown (D).

E, Scatter plots showing correlation analyses between percentages of CEBPB expression (x-axis) and percentages of immune cell infiltration markers CD68, CD163 and CD8 expression (y-axis) in 80 tumor samples from a TNBC-TMA analyzed by multiplex immunohistochemistry.

F, CEBPB-CKD plasmid structure model diagram and mRNA expression, protein expression of CEBPB in EMT6-CKD cells. *, $P < 0.05$, ***, $P < 0.001$.

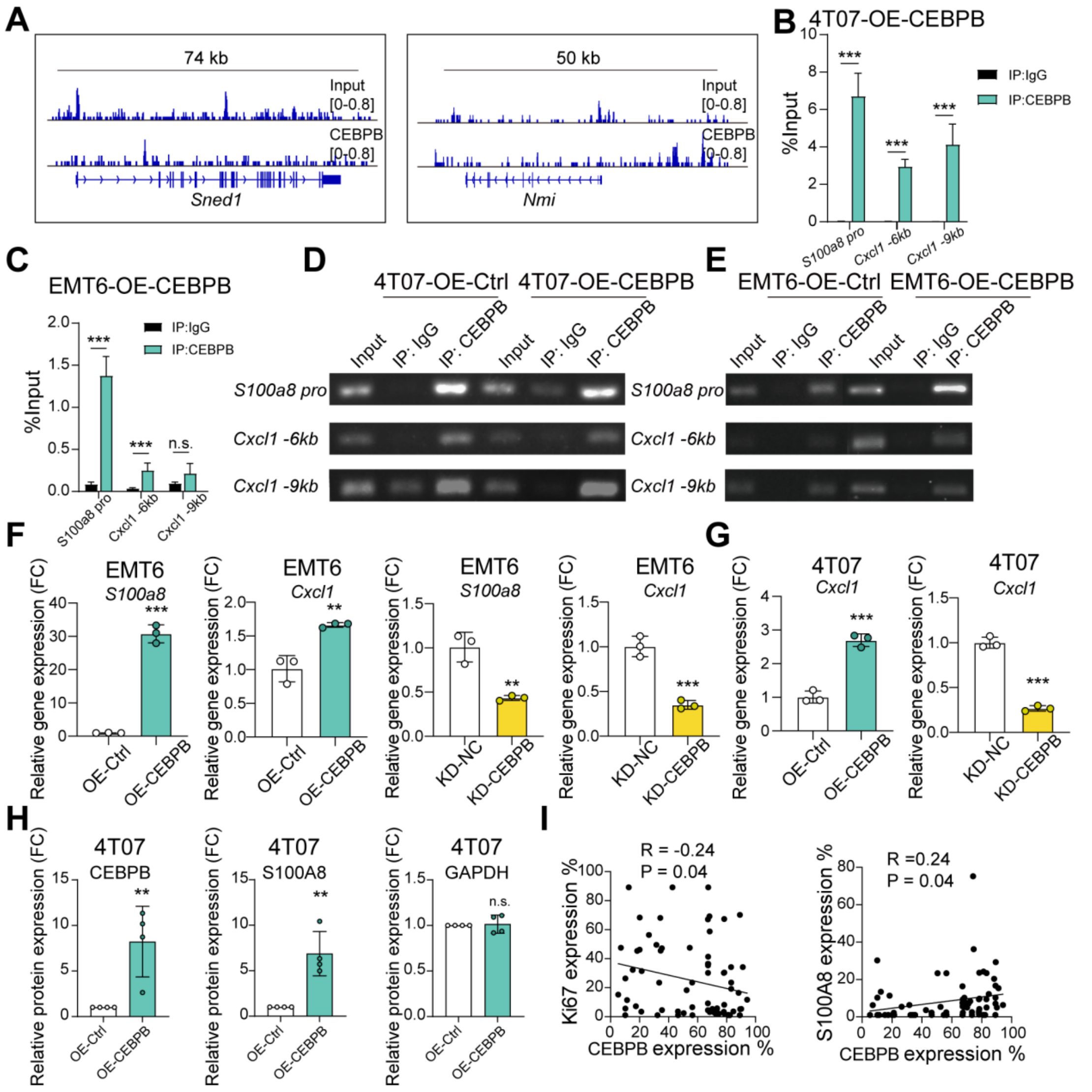

Figure S4. CEBPB transcriptionally activates S100A8 in TNBC.

A, CEBPB binding peaks for *Sned1* and *Nmi* from anti-CEBPB ChIP-seq in 4T07 cells. B-E, ChIP-qPCR (B-C) and DNA gel electrophoresis (D-E) showing the CEBPB binding regions in the *S100a8* promoter or upstream regions of *Cxcl1* of 4T07 and EMT6 cells with or without CEBPB overexpression.

F-G, mRNA expression of *S100a8* or *Cxcl1* in EMT6 (F) and 4T07 (G) cells with CEBPB overexpression or knockdown.

H, Corresponding statistic data of Figure6G.

I, Scatter plots showing correlation analyses between percentages of CEBPB expression (x-axis) and Ki67, and S100A8 expression (y-axis) in 80 tumor samples from a TNBC-TMA analyzed by multiplex immunohistochemistry. n.s., not significant, *, $P < 0.05$, **, $P < 0.01$, ***, $P < 0.001$.

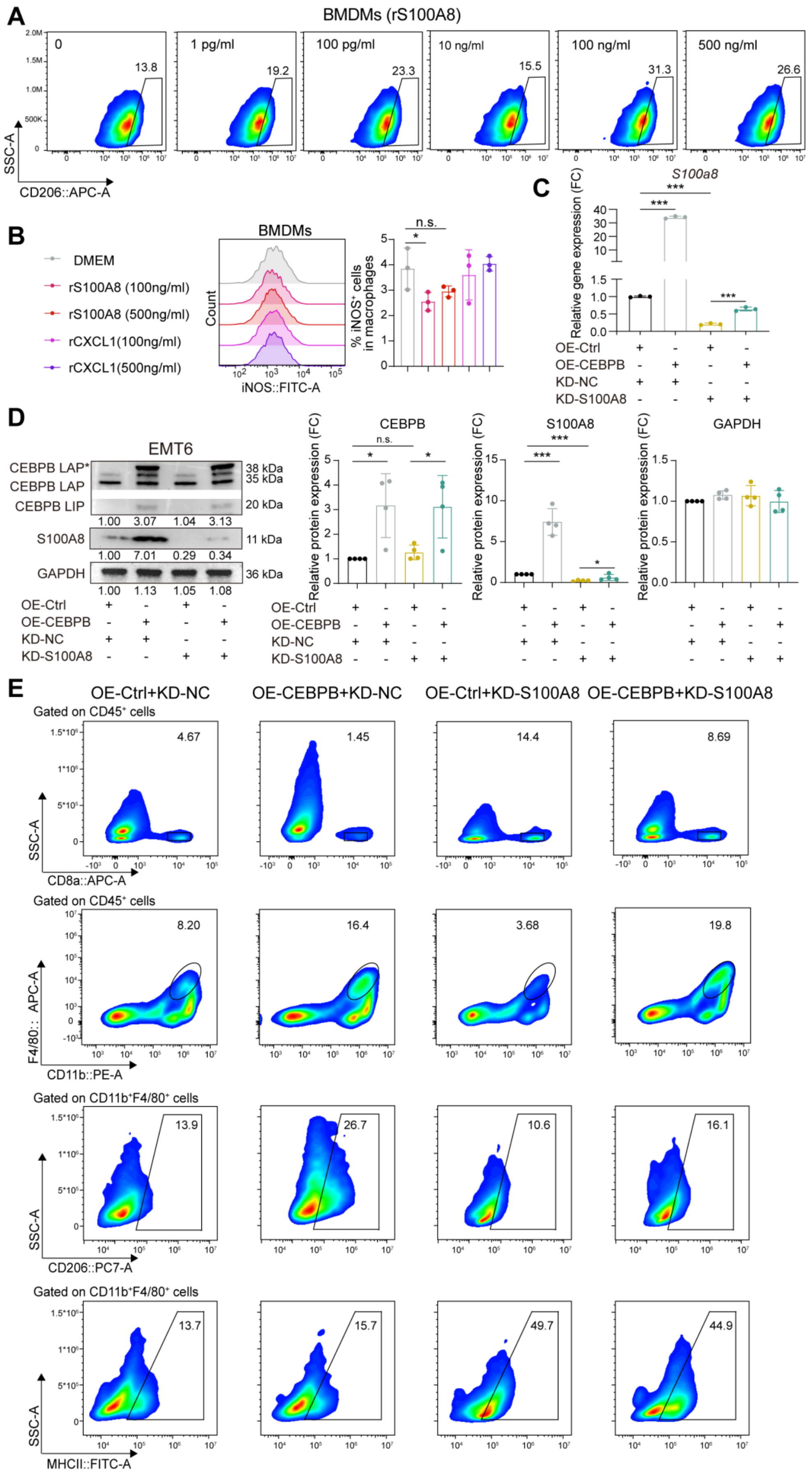
A
BMDMs (rS100A8)
0
1 pg/ml
100 pg/ml
10 ng/ml
100 ng/ml
500 ng/ml
13.8
19.2
23.3
15.5
31.3
26.6
SSC-A
CD206::APC-A
B
DMEM
rS100A8 (100ng/ml)
rS100A8 (500ng/ml)
rCXCL1(100ng/ml)
rCXCL1(500ng/ml)
BMDMs
Count
iNOS::FITC-A
% iNOS+ cells in macrophages
n.s.
C
S100a8
Relative gene expression (FC)
OE-Ctrl
OE-CEBPB
KD-NC
KD-S100A8
D
EMT6
CEBPB LAP*
CEBPB LAP
CEBPB LIP
38 kDa
35 kDa
20 kDa
1.00 3.07 1.04 3.13
S100A8
11 kDa
1.00 7.01 0.29 0.34
GAPDH
36 kDa
1.00 1.13 1.05 1.08
CEBPB
S100A8
GAPDH
Relative protein expression (FC)
E
OE-Ctrl+KD-NC
OE-CEBPB+KD-NC
OE-Ctrl+KD-S100A8
OE-CEBPB+KD-S100A8
Gated on CD45+ cells
4.67
1.45
14.4
8.69
SSC-A
CD8a::APC-A
Gated on CD45+ cells
8.20
16.4
3.68
19.8
F4/80:: APC-A
CD11b::PE-A
Gated on CD11b+F4/80+ cells
13.9
26.7
10.6
16.1
SSC-A
CD206::PC7-A
Gated on CD11b+F4/80+ cells
13.7
15.7
49.7
44.9
SSC-A
MHCII::FITC-A

Figure S5. knockdown S100A8 reverses immune evasion caused by CEBPB overexpression in TNBC.

A, Percentages of CD206 expressing BMDMs with the addition of rS100A8 at varying concentrations for 48 hours.

B, Percentage of iNOS expressing BMDMs with the addition of rS100A8 (100 or 500 ng/mL) or rCXCL1 (100 or 500 ng/mL) for 48 hours.

C-D, mRNA (C) and Protein (D) expression of CEBPB and S100A8 in EMT6 cells with CEBPB overexpression and/or S100A8 knockdown.

E, Flow cytometry analysis of $CD8^+$ T cells, $CD11b^+$ $F4/80^+$ cells, $CD206^+$ macrophages, and $MHCII^+$ macrophages infiltrated in tumors developed in CEBPB-overexpressing with or without S100A8 knockdown EMT6 allografts as in Figure 6L-R. *, $P < 0.05$, **, $P < 0.01$, ***, $P < 0.001$.

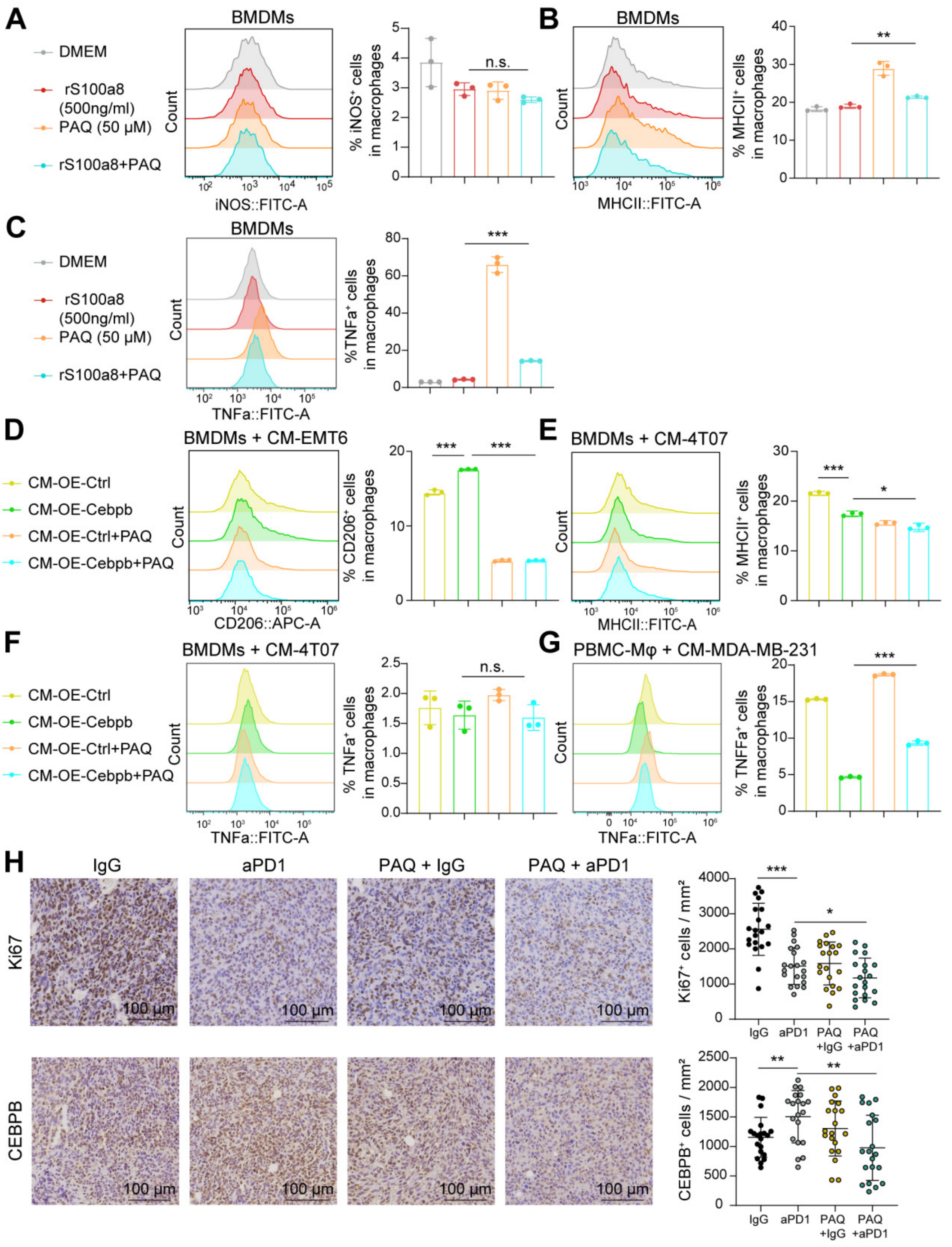

Figure S6. S100A8 inhibitor PAQ reverses macrophage M2 polarization caused by rS100a8 addition or CEBPB overexpression in TNBC.

A-C, Percentage of iNOS (A), MHC II (B) and TNFa (C) expressing BMDM with the addition of paquinimod (PAQ, 5 μg/mL) and/or rS100A8 (500 ng/mL) for 48 hours.

D, Percentages of CD206 expressing BMDM co-cultured with CM and/or PAQ (5 μg/mL) for 48 hours, including from EMT6 with or without CEBPB overexpression.

E, Percentages of MHCII expressing BMDM co-cultured with CM and/or PAQ (5 μg/mL) for 48 hours, including from 4T07 with or without CEBPB overexpression.

F-G, Percentages of TNFa expressing BMDM (F) or PMBC-Mφ (G) co-cultured with CM and/or PAQ (5 μg/mL) for 48 hours, including from 4T07 (F) or MDA-MB-231 (G) with or without CEBPB overexpression.

H, Representative immunohistochemistry images of Ki67 and CEBPB staining in tumor sections of EMT6 allografts in Figure.7F-H. *, $P < 0.05$, **, $P < 0.01$, ***, $P < 0.001$.
